# Supplementary material for: Examining therapeutic equivalence between branded and generic warfarin in Brazil: The WARFA crossover randomized controlled trial
Source: PLoS One. 2021 Apr 1;16(4):e0248567. doi: 10.1371/journal.pone.0248567 (PMC8016229; doi:10.1371/journal.pone.0248567)
Supplement: S1 Checklist — (PDF) [file pone.0248567.s001.pdf]

PDF-XChange Editor Click to BUY NOW! www.tracker-software.com

CONSORT 2010 checklist of information to include when reporting a randomised trial*

Section/Topic	Item No	Checklist item	Reported on page No
**Title and abstract**			
	1a	Identification as a randomised trial in the title	1
	1b	Structured summary of trial design, methods, results, and conclusions (for specific guidance see CONSORT for abstracts)	2
**Introduction**			
Background and objectives	2a	Scientific background and explanation of rationale	3
	2b	Specific objectives or hypotheses	3
**Methods**			
Trial design	3a	Description of trial design (such as parallel, factorial) including allocation ratio	3-4
	3b	Important changes to methods after trial commencement (such as eligibility criteria), with reasons	5,7-9 and S2 Appendix
Participants	4a	Eligibility criteria for participants	4-5
	4b	Settings and locations where the data were collected	4
Interventions	5	The interventions for each group with sufficient details to allow replication, including how and when they were actually administered	5
Outcomes	6a	Completely defined pre-specified primary and secondary outcome measures, including how and when they were assessed	6-7
	6b	Any changes to trial outcomes after the trial commenced, with reasons	7
Sample size	7a	How sample size was determined	7
	7b	When applicable, explanation of any interim analyses and stopping guidelines	N/A
Randomisation:			
Sequence generation	8a	Method used to generate the random allocation sequence	6
	8b	Type of randomisation; details of any restriction (such as blocking and block size)	6
Allocation concealment mechanism	9	Mechanism used to implement the random allocation sequence (such as sequentially numbered containers), describing any steps taken to conceal the sequence until interventions were assigned	6
Implementation	10	Who generated the random allocation sequence, who enrolled participants, and who assigned participants to interventions	6

PDF-XChange Editor
Click to BUY NOW!
www.tracker-software.com
PDF-XChange Editor
Click to BUY NOW!
www.tracker-software.com

Blin[illegible]g	11a	If done, who was blinded after assignment to interventions (for example, participants, care providers, those assessing outcomes) and how	6
	11b	If relevant, description of the similarity of interventions	6
Statistical methods	12a	Statistical methods used to compare groups for primary and secondary outcomes	7-9
	12b	Methods for additional analyses, such as subgroup analyses and adjusted analyses	8-9
**Results**			
Participant flow (a diagram is strongly recommended)	13a	For each group, the numbers of participants who were randomly assigned, received intended treatment, and were analysed for the primary outcome	10 and suppl. files (S2, S4 and S6 Figures)
	13b	For each group, losses and exclusions after randomisation, together with reasons	10 and suppl. files (S1-S6 Figures)
Recruitment	14a	Dates defining the periods of recruitment and follow-up	4
	14b	Why the trial ended or was stopped	7
Baseline data	15	A table showing baseline demographic and clinical characteristics for each group	11 and suppl. files (S5-S10 Tables)
Numbers analysed	16	For each group, number of participants (denominator) included in each analysis and whether the analysis was by original assigned groups	8-9 and suppl. files (S1-S6 Figures)
Outcomes and estimation	17a	For each primary and secondary outcome, results for each group, and the estimated effect size and its precision (such as 95% confidence interval)	13 and suppl. file (S1 Table)
	17b	For binary outcomes, presentation of both absolute and relative effect sizes is recommended	Suppl. file (S4 Table)
Ancillary analyses	18	Results of any other analyses performed, including subgroup analyses and adjusted analyses, distinguishing pre-specified from exploratory	14-15 and suppl. files (S1-S2 Table)
Harms	19	All important harms or unintended effects in each group (for specific guidance see CONSORT for harms)	Suppl. file (S4 Table)
**Discussion**			
Limitations	20	Trial limitations, addressing sources of potential bias, imprecision, and, if relevant, multiplicity of analyses	16,18
Generalisability	21	Generalisability (external validity, applicability) of the trial findings	17

PDF-XChange Editor Click to BUY NOW! www.tracker-software.com

Inter[illegible]etation	22	Interpretation consistent with results, balancing benefits and harms, and considering other relevant evidence	15-1[illegible]
**[illegible]er information**			
Registration	23	Registration number and name of trial registry	2-3
Protocol	24	Where the full trial protocol can be accessed, if available	3
Funding	25	Sources of funding and other support (such as supply of drugs), role of funders	1

*We strongly recommend reading this statement in conjunction with the CONSORT 2010 Explanation and Elaboration for important clarifications on all the items. If relevant, we also recommend reading CONSORT extensions for cluster randomised trials, non-inferiority and equivalence trials, non-pharmacological treatments, herbal interventions, and pragmatic trials. Additional extensions are forthcoming: for those and for up to date references relevant to this checklist, see www.consort-statement.org.

PDF-XChange Editor Click to BUY NOW! www.tracker-software.com

PDF-XChange Editor Click to BUY NOW! www.tracker-software.com
PDF-XChange Editor Click to BUY NOW! www.tracker-software.com

Table 1
CONSORT checklist of information to include when reporting randomised crossover trials

Section/topic	Item No	Description	Page No
Title	1a	Identification as a randomised crossover trial in the title	1
Abstract	1b	Specify a crossover design and report all information outlined in table 2	2
Introduction:			
Background	2a	Scientific background and explanation of rationale	3
Objectives	2b	Specific objectives or hypotheses	3
Methods:			
Trial design	3a	Rationale for a crossover design. Description of the design features including allocation ratio, especially the number and duration of periods, duration of washout period, and consideration of carry over effect	3-4
Change from protocol	3b	Important changes to methods after trial commencement (such as eligibility criteria), with reasons	5,7-9 and S2 Appendix
Participants	4a	Eligibility criteria for participants	4-5
Settings and location	4b	Settings and locations where the data were collected	4
Interventions	5	The interventions with sufficient details to allow replication, including how and when they were actually administered	5
Outcomes	6a	Completely defined prespecified primary and secondary outcome measures, including how and when they were assessed	6-7
Changes to outcomes	6b	Any changes to trial outcomes after the trial commenced, with reasons	7
Sample size	7a	How sample size was determined, accounting for within participant variability	7
Interim analyses and stopping guidelines	7b	When applicable, explanation of any interim analyses and stopping guidelines	N/A
Randomisation:			
Sequence generation	8a	Method used to generate the random allocation sequence	6
Sequence generation	8b	Type of randomisation; details of any restriction (such as blocking and block size)	6
Allocation concealment mechanism	9	Mechanism used to implement the random allocation sequence (such as sequentially numbered containers), describing any steps taken to conceal the sequence until interventions were assigned	6
Implementation	10	Who generated the random allocation sequence, who enrolled participants, and who assigned participants to the sequence of interventions	6

PDF-XChange Editor Click to BUY NOW! www.tracker-software.com

PDF-XChange Editor Click to BUY NOW! www.tracker-software.com

Section/topic	Item No	Description	Page No
Blinding	11a	If done, who was blinded after assignment to interventions (for example, participants, care providers, those assessing outcomes) and how	6
Similarity of interventions	11b	If relevant, description of the similarity of interventions	6
Statistical methods	12a	Statistical methods used to compare groups for primary and secondary outcomes which are appropriate for crossover design (that is, based on within participant comparison)	7-9
Additional analyses	12b	Methods for additional analyses, such as subgroup analyses and adjusted analyses	8-9
Results			
Participant flow (a diagram is strongly recommended)	13a	The numbers of participants who were randomly assigned, received intended treatment, and were analysed for the primary outcome, separately for each sequence and period	10 and suppl. files (S2, S4 and S6 Figures)
Losses and exclusions	13b	No of participants excluded at each stage, with reasons, separately for each sequence and period	10 and suppl. files (S1-S6 Figures)
Recruitment	14a	Dates defining the periods of recruitment and follow-up	4
Trial end	14b	Why the trial ended or was stopped	7
Baseline data	15	A table showing baseline demographic and clinical characteristics by sequence and period	Suppl. files (S5-S10 Tables)
Numbers analysed	16	Number of participants (denominator) included in each analysis and whether the analysis was by original assigned groups	8-9 and suppl. files (S1-S6 Figures)
Outcomes and estimation	17a	For each primary and secondary outcome, results including estimated effect size and its precision (such as 95% confidence interval) should be based on within participant comparisons. In addition, results for each intervention in each period are recommended	13 and suppl. file (S1 Table)
Binary outcomes	17b	For binary outcomes, presentation of both absolute and relative effect sizes is recommended	Suppl. file (S4 Table)
Ancillary analyses	18	Results of any other analyses performed, including subgroup analyses and adjusted analyses,	14-15 and

PDF-XChange Editor Click to BUY NOW! www.tracker-software.com

Section/topic	Item No	Description	Page No
		distinguishing prespecified from exploratory	suppl. files (S1-S2 Table)
Harms	19	Describe all important harms or untended effects in a way that accounts for the design (for specific guidance, see CONSORT for harms)	Suppl. file (S4 Table)
Discussion:			
Limitations	20	Trial limitations, addressing sources of potential bias, imprecision, and if relevant, multiplicity of analyses. Consider potential carry over effects	16,18
Generalisability	21	Generalisability (external validity, applicability) of the trial findings	17
Interpretation	22	Interpretation consistent with results, balancing benefits and harms, and considering other relevant evidence	15-19
Other information:			
Registration	23	Registration number and name of trial registry	2-3
Protocol	24	Where the full trial protocol can be accessed, if available	3
Funding	25	Sources of funding and other support (such as supply of drugs), role of funders	1

CONSORT=Consolidated Standards of Reporting Trials.
