# Supplementary figures and images for: Examining therapeutic equivalence between branded and generic warfarin in Brazil: The WARFA crossover randomized controlled trial
Source: PLoS One. 2021 Apr 1;16(4):e0248567. doi: 10.1371/journal.pone.0248567 (PMC8016229; doi:10.1371/journal.pone.0248567)

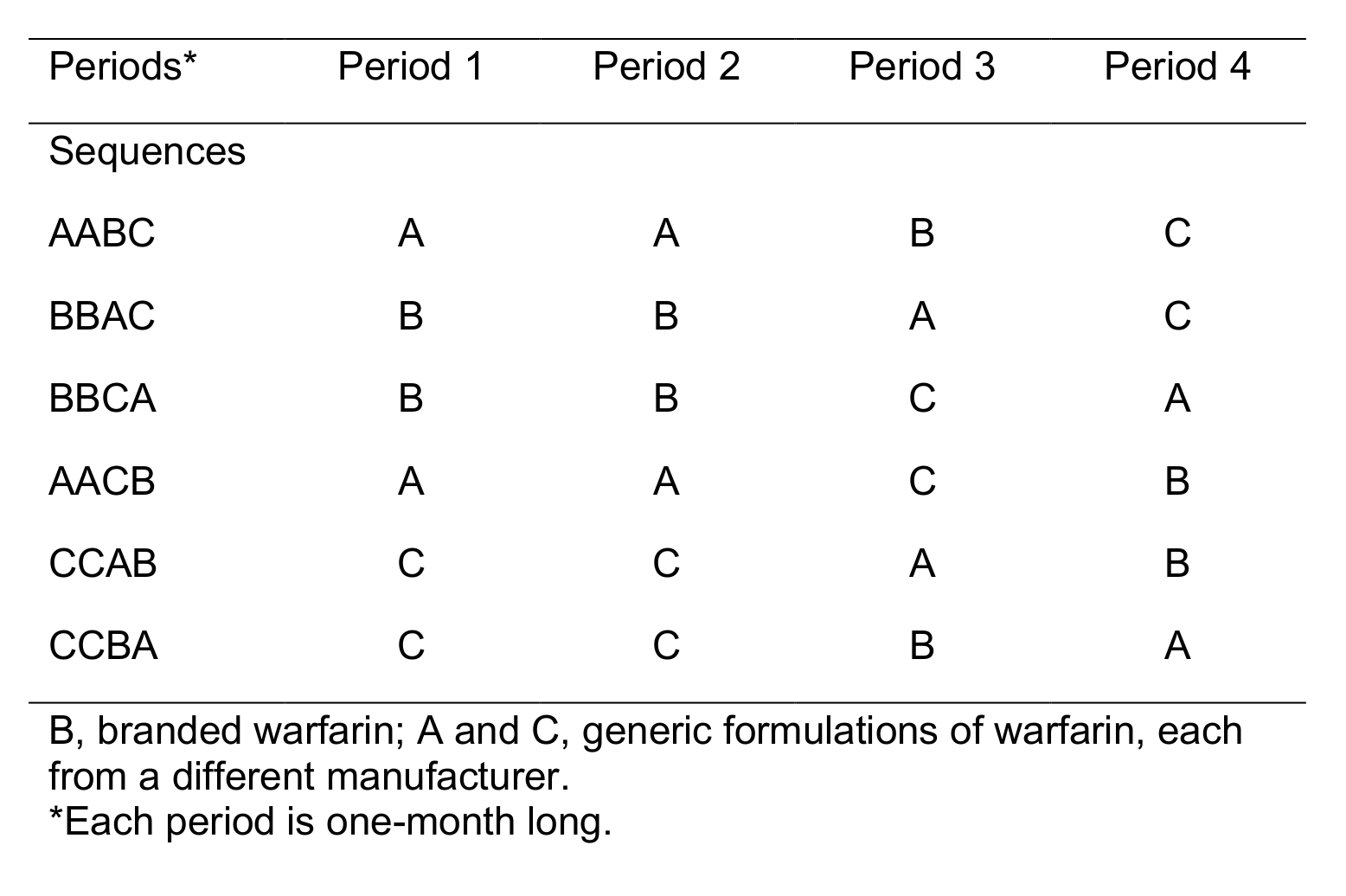

Supplement: S1 Fig — B, branded warfarin; A and C, generic formulations of warfarin, each from a different manufacturer. *Each period duration is one month. Republished with minor alterations from [8] under a CC BY license (http://creativecommons.org/licenses/by/4.0/), with permission from BioMed Central, original copyright 2017. (TIF) [file pone.0248567.s002.tif]

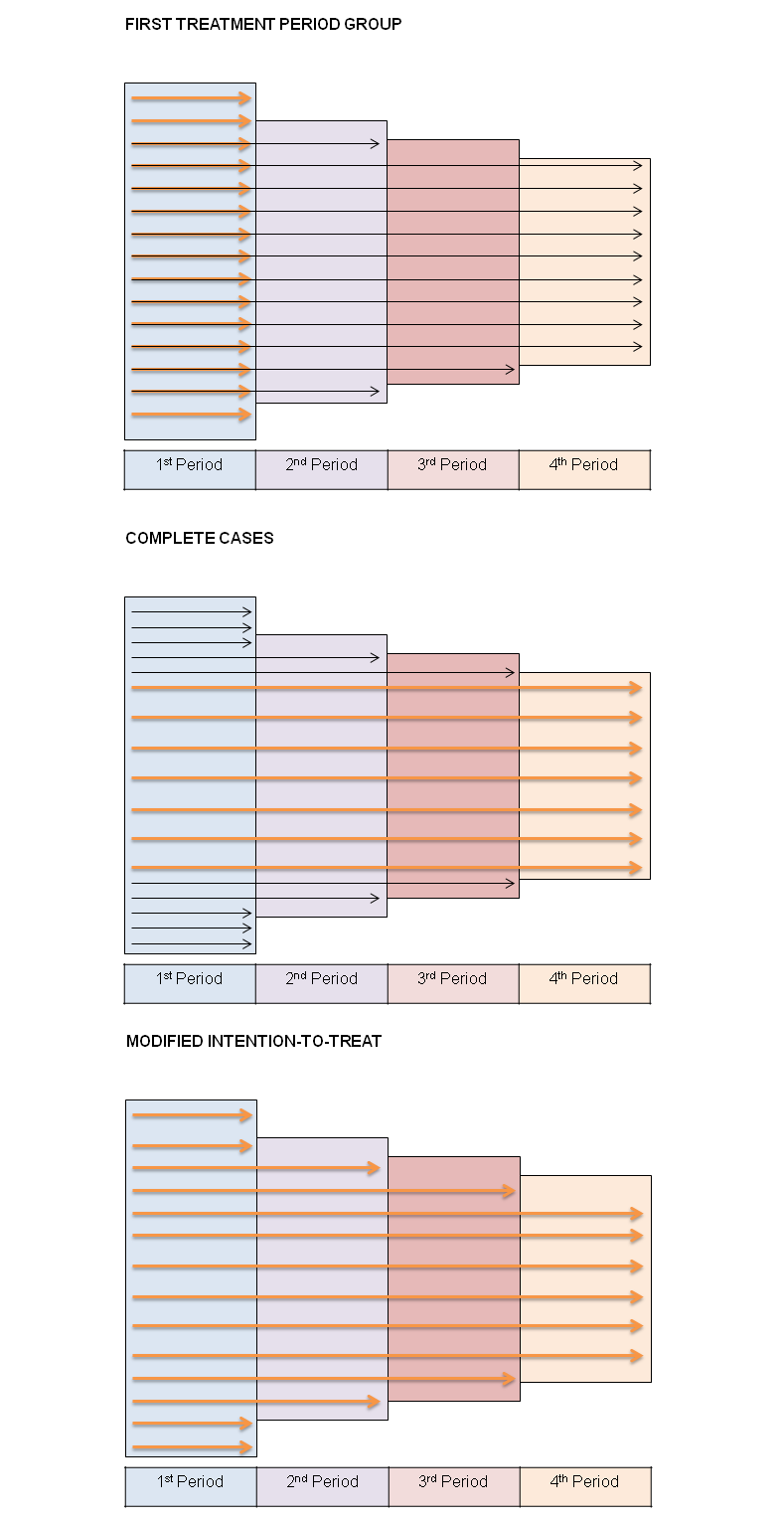

Supplement: S2 Fig — A) First treatment period group population, which included only data of the first period for participants with at least one valid INR or ΔINR in this same period. B) Complete cases population, which comprised data from patients that had at least one valid INR or ΔINR in every treatment period. C) Modified intention-to-treat population, which comprised participants with at least one valid INR or ΔINR in any time period. The arrows represent data collected from study subjects throughout the trial; each arrow represents one participant. The orange arrows represent the data from each patient that was included in that population for analysis. (TIFF) [file pone.0248567.s003.tiff]
