# Supplementary material for: Examining therapeutic equivalence between branded and generic warfarin in Brazil: The WARFA crossover randomized controlled trial
Source: PLoS One. 2021 Apr 1;16(4):e0248567. doi: 10.1371/journal.pone.0248567 (PMC8016229; doi:10.1371/journal.pone.0248567)
Supplement: S3 Fig — (PDF) [file pone.0248567.s004.pdf]

S3 Fig. Flow diagram of the participants of the WARFA trial, by sequence and period, for the subpopulation First treatment period group and the outcomes of mean INR and mean warfarin dose per week.

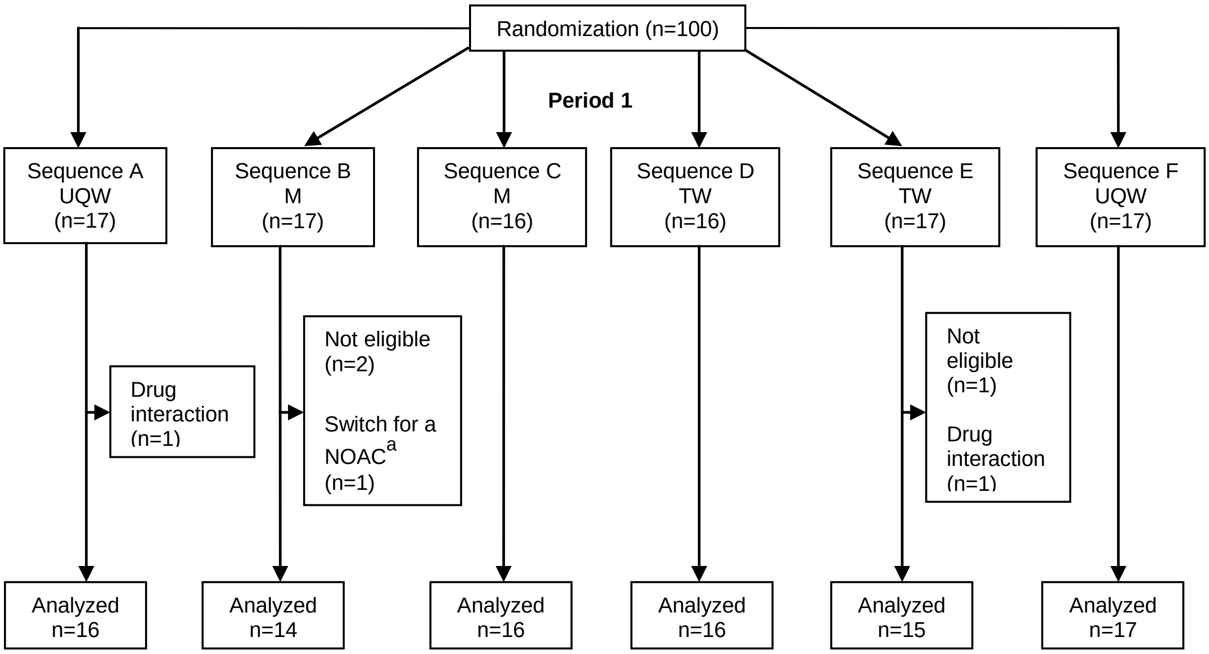

M: Marevan; TW: Teuto warfarin; UQW: União Química warfarin; INR: international normalized ratio; NOAC: novel anticoagulant. To be included in the analysis for these outcomes, patients needed at least one valid INR value, either from the third or fourth weeks of the study.

[a] Warfarin switched for a NOAC due to arrhythmia ablation procedures and not because of adverse events.
