# Supplementary material for: Examining therapeutic equivalence between branded and generic warfarin in Brazil: The WARFA crossover randomized controlled trial
Source: PLoS One. 2021 Apr 1;16(4):e0248567. doi: 10.1371/journal.pone.0248567 (PMC8016229; doi:10.1371/journal.pone.0248567)
Supplement: S4 Fig — (PDF) [file pone.0248567.s005.pdf]

S4 Fig. Flow diagram of the participants of the WARFA trial, by sequence and period, for the subpopulation First treatment period group and the outcomes of ΔINR, Δ dose, and mean TTR.

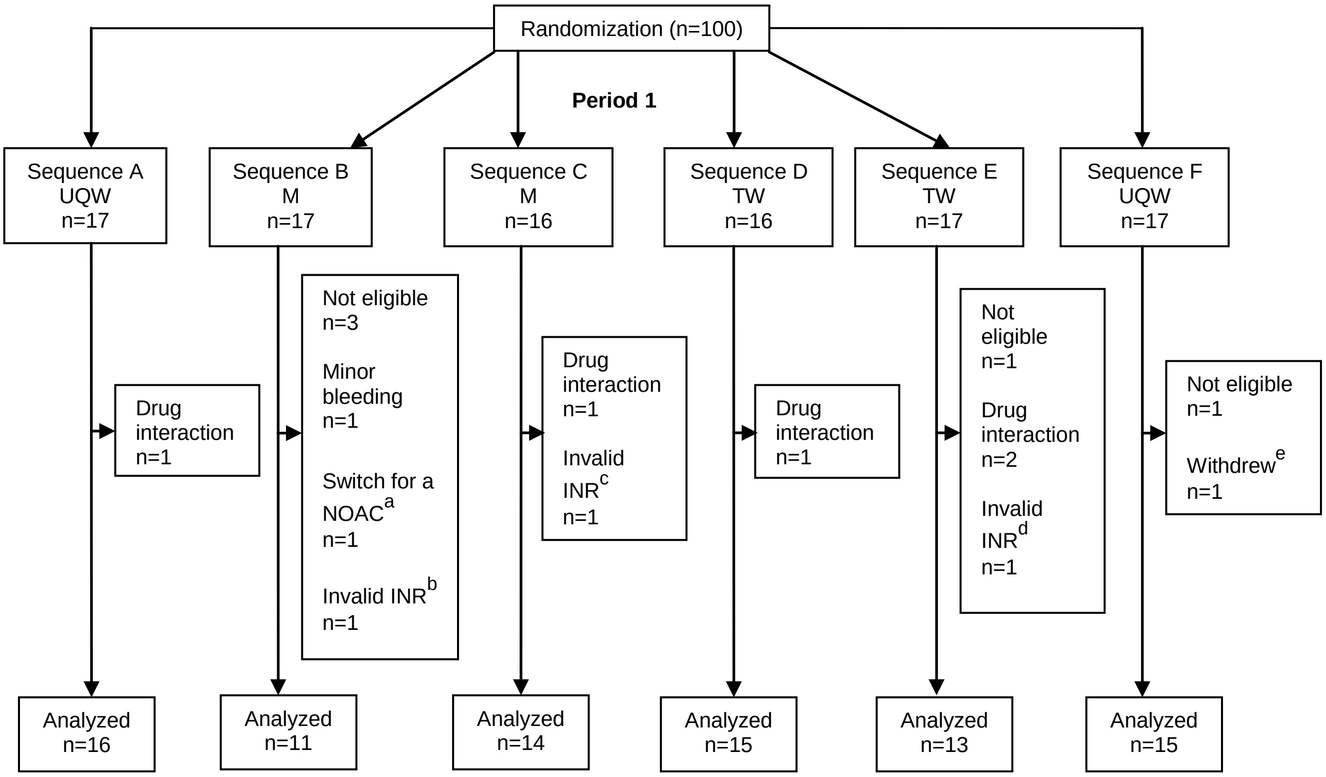

M: Marevan; TW: Teuto warfarin; UQW: União Química warfarin; INR: international normalized ratio; NOAC: novel anticoagulant. To be included in the analysis for these outcomes, patients needed to have two valid INR values, at the third and fourth weeks of the study.

[a] Warfarin switched for a NOAC due to arrhythmia ablation procedures and not because of adverse events.

[b] Patient excluded from the analysis due to a diarrhea in the day of the fourth week test and in the previous day.

[c] Patient excluded from the analysis because she had diarrhea in the three days prior to the fourth week test. In addition, she did not take warfarin in the days she was unwell.

[d] Patient excluded from the analysis because he had stopped taking warfarin (with no apparent reason) for the three days prior to the fourth week test.

[e] Patient withdrew due to study visits not fitting into his personal schedule.
