# Supplementary material for: Examining therapeutic equivalence between branded and generic warfarin in Brazil: The WARFA crossover randomized controlled trial
Source: PLoS One. 2021 Apr 1;16(4):e0248567. doi: 10.1371/journal.pone.0248567 (PMC8016229; doi:10.1371/journal.pone.0248567)
Supplement: S5 Fig — (PDF) [file pone.0248567.s006.pdf]

S5 Fig. Flow diagram of the participants of the WARFA trial, by sequence and period, for the subpopulation Modified intention-to-treat and the outcomes of mean INR and mean warfarin dose per week.

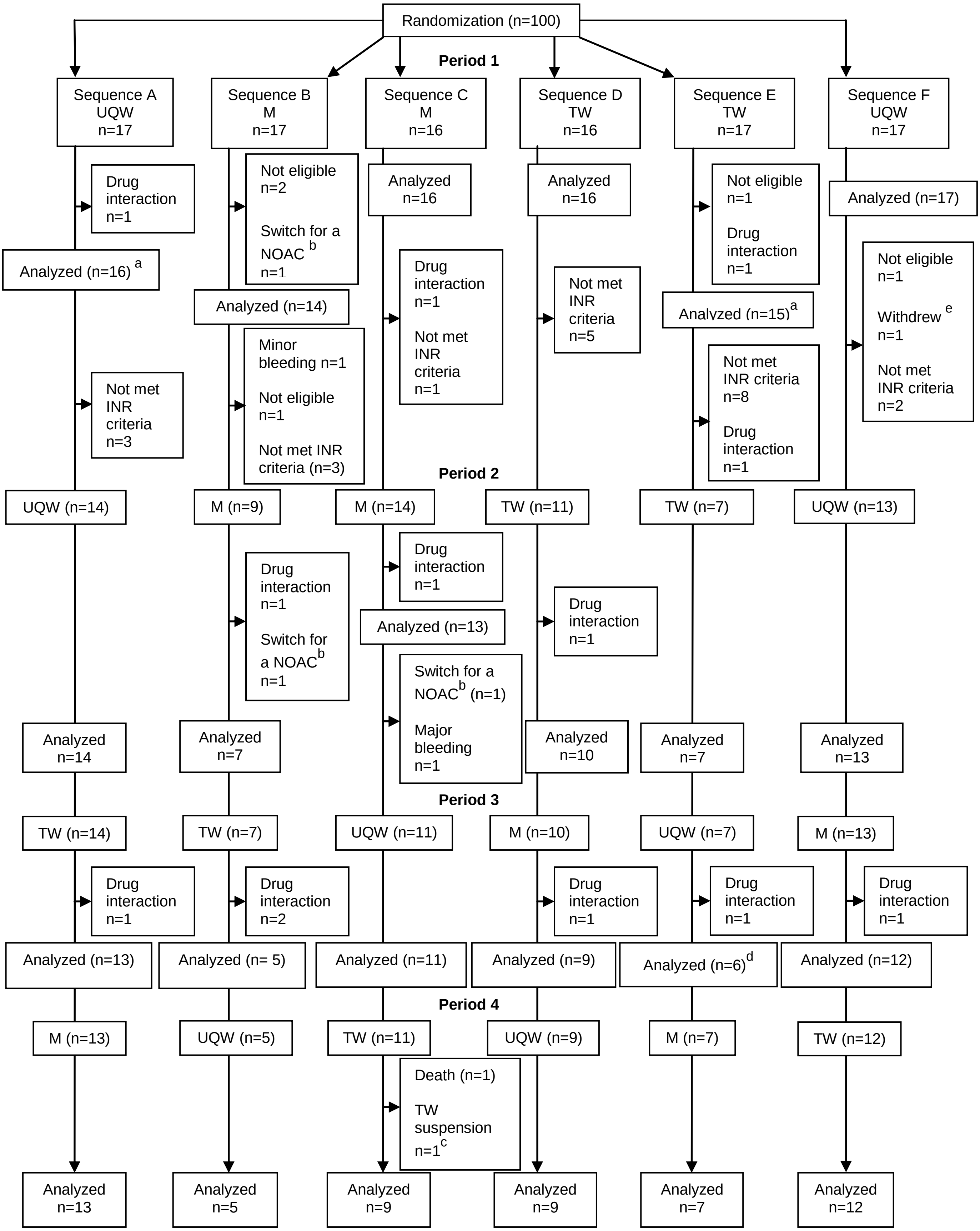

M: Marevan; TW: Teuto warfarin; UQW: União Química warfarin; INR: international normalized ratio; NOAC: novel anticoagulant. The multilevel mixed-effects linear regression models with individuals as random intercepts allowed us to include in this analysis even patients that did not have outcome results in every study period.

[a] One patient not included in the analysis due to drug interaction. The same patient was later excluded from further study periods due to not meeting INR criteria.

[b] Warfarin replaced by a NOAC due to arrhythmia ablation procedures and not because of adverse events.

[c] Patient developed hypersensitivity type I reaction to TW and thus was switched back to UQW.

[d] Patient's outcome for this period was not included in the analysis because of an acute drug interaction with warfarin.
[e] Patient withdrew due to study visits not fitting into his personal schedule.
