# Supplementary material for: Examining therapeutic equivalence between branded and generic warfarin in Brazil: The WARFA crossover randomized controlled trial
Source: PLoS One. 2021 Apr 1;16(4):e0248567. doi: 10.1371/journal.pone.0248567 (PMC8016229; doi:10.1371/journal.pone.0248567)
Supplement: S6 Fig — (PDF) [file pone.0248567.s007.pdf]

S6 Fig. Flow diagram of the participants of the WARFA trial, by sequence and period, for the subpopulation Modified intention-to-treat and the outcomes of ∆INR, ∆ dose and mean TTR.

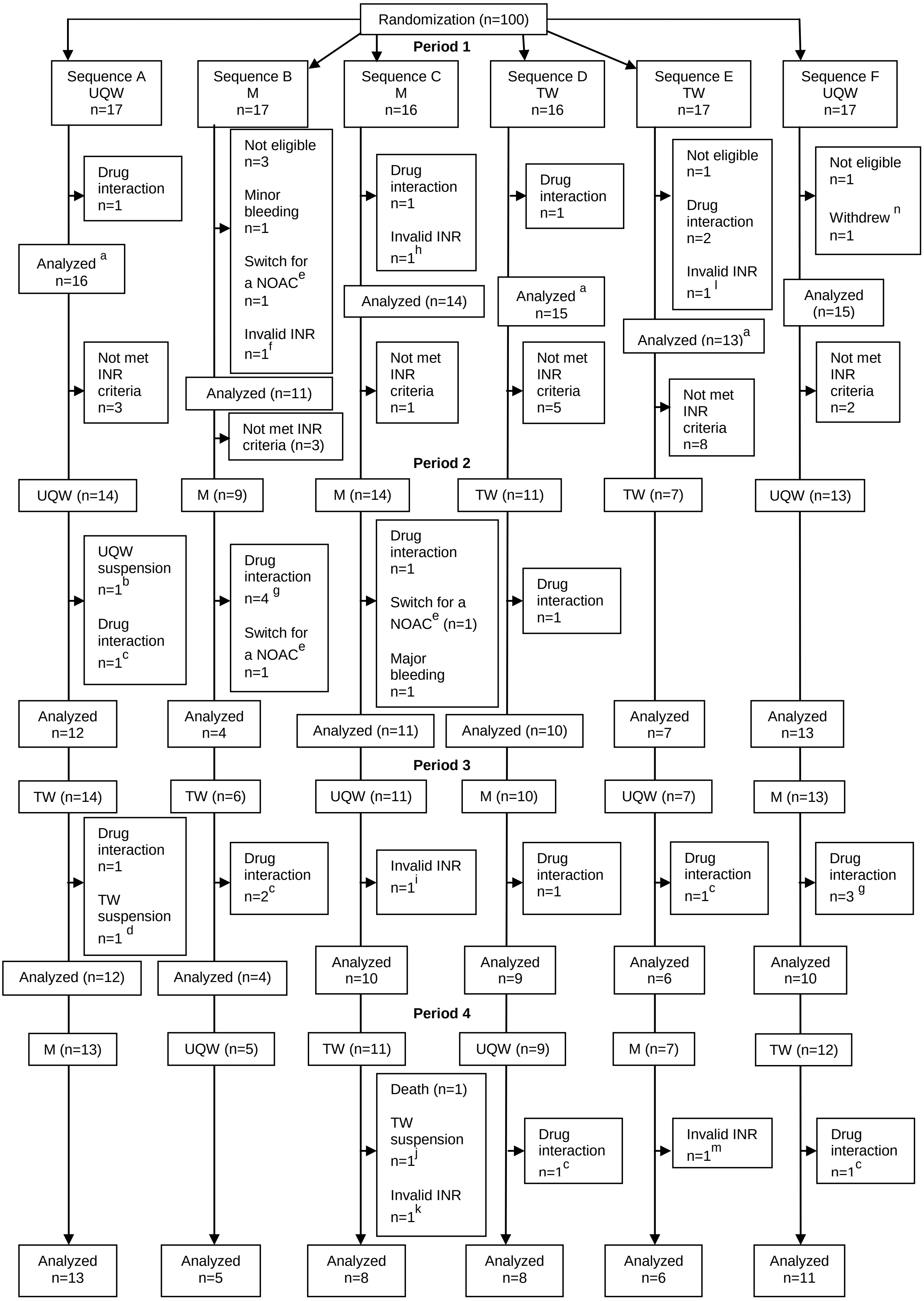

M: Marevan; TW: Teuto warfarin; UQW: União Química warfarin; INR: international normalized ratio; NOAC: novel anticoagulant. The multilevel mixed-effects linear regression models with individuals as random intercepts allowed us to include in this analysis even patients that did not have outcome results in every study period.

[a] One patient not included in the analysis due to drug interaction. The same patient was later excluded from further study periods due to not meeting INR criteria.

[b] Patient's outcome for this period was not included in the analysis because he stopped taking warfarin for four days prior to the seventh week test, in preparation for an endoscopy.

[c] Patient's outcome for this period was not included in the analysis because of an acute drug interaction with warfarin.

[d] Patient's outcome for this period was not included in the analysis because she did not take warfarin for ten days, a week prior to the eleventh week test, while as an inpatient for an unrelated health issue.

[e] Warfarin replaced by a NOAC due to arrhythmia ablation procedures and not because of adverse events.

[f] Patient's outcome for this period was not included in the analysis due to a diarrhea in the day and in the previous day of the fourth week test.

[g] Two patients' outcomes for this period were not included in the analysis because of acute drug interactions with warfarin.

[h] Patient's outcome for this period was not included in the analysis because she had diarrhea in the three days prior to the fourth week test. In addition, she did not take warfarin in the days she was unwell.

[i] Patient's outcome for this period was not included in the analysis because she stopped taking simvastatin, medication that interacts with warfarin, for 9-14 days prior to the eleventh week test. After orientation, patient resumed treatment with simvastatin.

[j] Patient developed hypersensitivity type I reaction to TW and thus was switched back to UQW.

[k] Patient's outcome for this period was not included in the analysis because she had stopped taking warfarin for four days prior to the sixteenth week test due to a dermatologic procedure.

[l] Patient's outcome for this period was not included in the analysis because he had stopped taking warfarin (with no apparent reason) for the three days prior to the fourth week test.

[m] Patient's outcome for this period was not included in the analysis because he had diarrhea in the two days prior to the fifteenth week test.

[n] Patient withdrew due to study visits not fitting into his personal schedule.
