# Supplementary material for: Examining therapeutic equivalence between branded and generic warfarin in Brazil: The WARFA crossover randomized controlled trial
Source: PLoS One. 2021 Apr 1;16(4):e0248567. doi: 10.1371/journal.pone.0248567 (PMC8016229; doi:10.1371/journal.pone.0248567)
Supplement: S7 Fig — (PDF) [file pone.0248567.s008.pdf]

S7 Fig. Flow diagram of the participants of the WARFA trial, by sequence and period, for the subpopulation Complete cases and the outcomes of mean INR and mean warfarin dose per week.

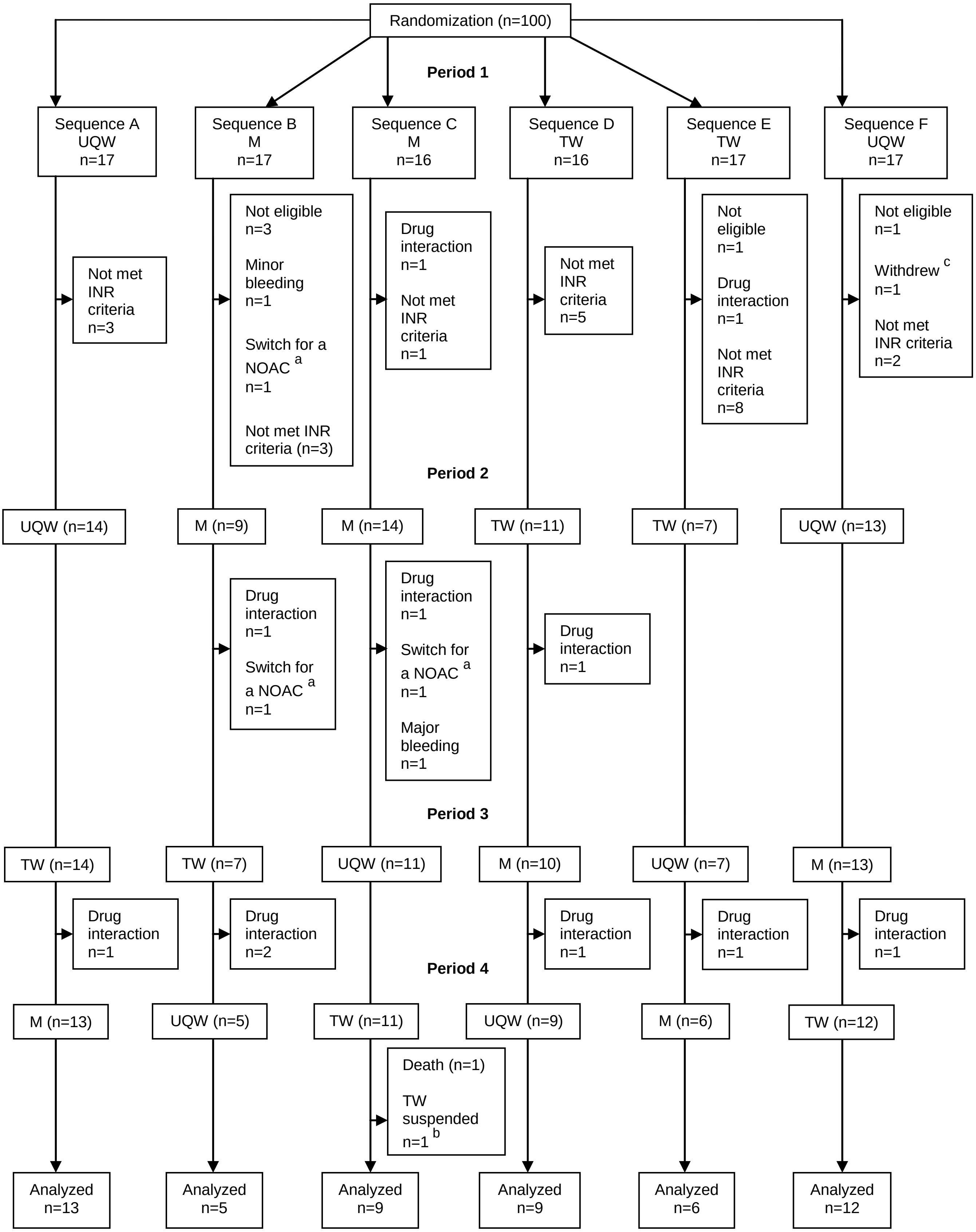

M: Marevan; TW: Teuto warfarin; UQW: União Química warfarin; INR: international normalized ratio; NOAC: novel anticoagulant.

[a] Warfarin switched for a NOAC due to arrhythmia ablation procedures and not because of adverse events.
[b] Patient developed hypersensitivity type I reaction to TW and decided to switch back to UQW.
[c] Patient withdrew due to study visits not fitting into his personal schedule.
