# Supplementary material for: Examining therapeutic equivalence between branded and generic warfarin in Brazil: The WARFA crossover randomized controlled trial
Source: PLoS One. 2021 Apr 1;16(4):e0248567. doi: 10.1371/journal.pone.0248567 (PMC8016229; doi:10.1371/journal.pone.0248567)
Supplement: S8 Fig — (PDF) [file pone.0248567.s009.pdf]

S8 Fig. Flow diagram of the participants of the WARFA trial, by sequence and period, for the subpopulation Complete cases and the outcomes of ∆INR, ∆ dose, and mean TTR.

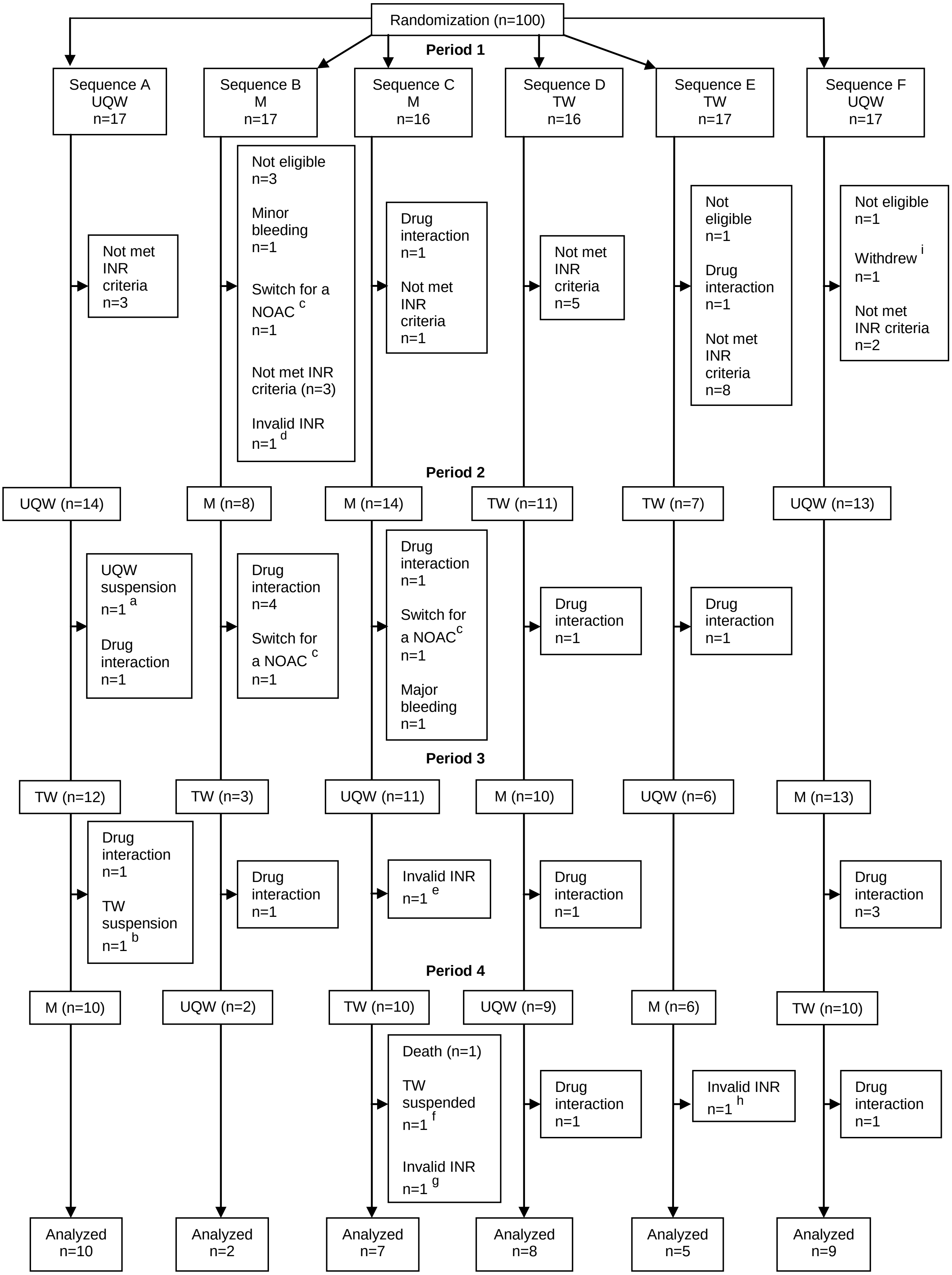

M: Marevan; TW: Teuto warfarin; UQW: União Química warfarin; INR: international normalized ratio; NOAC: novel anticoagulant. To be included in the analysis for these outcomes, patients needed to have two valid INR values in each one of the four periods of the trial.

[a] Patient stopped taking warfarin for four days prior to the seventh week test, in preparation for an endoscopy.

[b] Patient did not take warfarin for ten days, a week prior to the eleventh week test, while as an inpatient for an unrelated health issue.

[c] Warfarin switched for a NOAC due to arrhythmia ablation procedures and not because of adverse events.

[d] Patient had a diarrhea in the day and in the previous day of the fourth week test.

[e] Patient stopped taking simvastatin, medication that interacts with warfarin, for 9-14 days prior to the eleventh week test.

[f] Patient developed hypersensitivity type I reaction to TW and decided to switch back to UQW.

[g] Patient's outcome for this period was not included in the analysis because she had stopped taking warfarin for four days prior to the sixteenth week test due to a dermatologic procedure.

[h] Patient had diarrhea in the two days prior to the fifteenth week test.

[i] Patient withdrew due to study visits not fitting into his personal schedule.
