# Supplementary material for: Examining therapeutic equivalence between branded and generic warfarin in Brazil: The WARFA crossover randomized controlled trial
Source: PLoS One. 2021 Apr 1;16(4):e0248567. doi: 10.1371/journal.pone.0248567 (PMC8016229; doi:10.1371/journal.pone.0248567)
Supplement: S1 Table — (PDF) [file pone.0248567.s010.pdf]

S1 Table. Outcomes of the WARFA trial for the Complete cases and the Modified intention-to-treat populations.

**Outcome**	**Differences between UQW and Marevan** Mean (95%CI)	**Differences between TW and Marevan** Mean (95%CI)	**Differences between TW and UQW** Mean (95%CI)	**n**
**Complete cases population**				
Δ INR[a]	-	-	-	-
INR	-0.15 (-0.29 to -0.01)	-0.12 (-0.26 to +0.03)	+0.03 (-0.10 to +0.18)	54
Dose (mg) per week[b]	+0.4 (0.0 to +0.9)	-0.2 (-0.8 to +0.3)	-0.7 (-1.2 to -0.2)	54
Δ dose (mg)	-0.7 (-1.5 to 0.0)	-0.6 (-1.3 to +0.1)	+0.1 (-0.5 to +0.8)	41
TTR (%)[c]	+13.7 (-2.4 to +29.9)	+5.7 (-10.8 to +22.2)	-8.0 (-23.1 to +7.0)	33
**Modified intention-to-treat population**				
Δ INR[d]	-	-	-	-
INR	-0.14 (-0.28 to +0.01)	-0.06 (-0.20 to +0.09)	+0.08 (-0.06 to +0.22)	94
Dose (mg) per week	+0.2 (-0.2 to +0.6)	0.0 (-0.4 to +0.4)	-0.2 (-0.6 to +0.2)	94
Δ dose (mg)	-0.8 (-1.4 to -0.1)	-0.4 (-1.0 to +0.3)	+0.4 (-0.2 to +1.1)	84
TTR (%)[c]	+11.8 (-1.7 to +25.3)	-4.3 (-18.0 to +9.3)	-16.1 (-28.6 to -3.7)	71

CI: confidence interval; ΔINR: INR variability; INR: international normalized rate; TW: Teuto warfarin; TTR: time in therapeutic range; UQW: União Química warfarin.

[a] Not calculated due to period effects. After exclusion of data from the 3rd period due to period effects (P=0.005), the 2nd and the 4th periods also turned significant (P=0.025 and P=0.06, respectively), thus we did not pool the observations from different periods.

[b] After exclusion of the data from the 4th period due to period effects (P=0.010).

[c] After exclusion of the data from the C sequence due to sequence effects (P=0.039 and P=0.027 for the Complete cases and the Modified intention-to-treat populations, respectively).

[d] Not calculated due to period effects. After exclusion of the data from the 3rd period due to period effects (P=0.012), the 4th period also turned significant (P=0.020) and after exclusion of the data from the latter, treatments were automatically omitted from the model due to collinearity. Collinearity of variables means the variables are nearly the same, in a way that is difficult to estimate their separate effects.
