# Supplementary material for: Examining therapeutic equivalence between branded and generic warfarin in Brazil: The WARFA crossover randomized controlled trial
Source: PLoS One. 2021 Apr 1;16(4):e0248567. doi: 10.1371/journal.pone.0248567 (PMC8016229; doi:10.1371/journal.pone.0248567)
Supplement: S2 Table — (PDF) [file pone.0248567.s011.pdf]

S2 Table. Results for analyses of continuous outcomes of the WARFA trial by the bootstrap method (100 replicates).

Outcome	n	Differences between UQW and Marevan Mean (95%CI)	Differences between TW and Marevan Mean (95%CI)	Differences between TW and UQW Mean (95%CI)
**First treatment period group**				
∆ INR	84	+0.09 (-0.17 to +0.35)	+0.29 (-0.06 to +0.64)	+0.20 (-0.18 to +0.59)
INR	94	-0.05 (-0.23 to +0.12)	+0.23 (0.00 to +0.48)	+0.29 (+0.03 to +0.55)
Dose (mg) per week[a]	94	+0.6 (-0.1 to +1.3)	+0.9 (-0.2 to +1.9)	+0.2 (-0.4 to +0.9)
∆ dose (mg)	83	-1.2 (-2.6 to +0.2)	+0.1 (-1.7 to +1.9)	+1.3 (0.0 to +2.5)
TTR (%)	84	+3.7 (-19.9 to +27.4)	-24.5 (-47.6 to -1.4)	-28.2 (-48.8 to -7.6)
**Complete cases population**				
∆ INR[b]	-	-	-	-
INR	54	-0.15 (-0.29 to -0.01)	-0.12 (-0.29 to +0.06)	+0.03 (-0.10 to +0.17)
Dose (mg) per week[c]	-	-	-	-
∆ dose (mg)	41	-0.7 (-1.6 to +0.1)	-0.6 (-1.4 to +0.2)	+0.1 (-0.4 to +0.7)
TTR (%)[d]	33	+13.7 (-2.9 to +30.3)	+5.7 (-11.0 to +22.4)	-8.0 (-20.5 to +4.4)
**Modified intention-to-treat population**				
∆ INR[e]	-	-	-	-
INR[f]	78	-0.14 (-0.29 to 0.00)	-0.12 (-0.29 to +0.06)	+0.02 (-0.16 to +0.20)
Dose per week (mg)[g]	45	+0.3 (-0.4 to +0.9)	-0.5 (-1.2 to +0.2)	-0.7 (-1.2 to -0.3)
∆ dose (mg)	84	-0.8 (-1.6 to 0.0)	-0.4 (-1.2 to +0.4)	+0.4 (-0.1 to +0.9)
TTR (%)[d]	71	+11.8 (-1.1 to +24.7)	-4.3 (-17.2 to +8.5)	-16.1 (-28.4 to -3.9)

CI: confidence interval; ∆INR: INR variability; INR: international normalized rate; UQW: União Química warfarin; TW: Teuto warfarin; TTR: time in therapeutic range.

[a] Bootstrap applied to the multiple linear regression model adjusted by the baseline dose.

[b] Bootstrap not applied because the original analysis was affected by period effects.

[c] Not calculated due to period and sequence effects. After exclusion of the data from the 4th period due to period effects, the bootstrap simulation resulted in sequences C, D, E and F significant (all with P < 0.001). After exclusion of the data from these sequences, treatments were automatically omitted from the model due to collinearity.

[e] Bootstrap not applied because the original analysis was affected by period effects.

[d] Bootstrap applied to the mixed model that did not include data from the C sequence.

[f] Bootstrap applied to the mixed model that did not include data from the D sequence (sequence effects from sequence D, P < 0.05).

[g] Bootstrap applied to the mixed model that did not include data from sequences C, D and F (sequence effects from C sequence, P = 0.038; D sequence, P < 0.001, and F sequence, P = 0.006).
