# Supplementary material for: Examining therapeutic equivalence between branded and generic warfarin in Brazil: The WARFA crossover randomized controlled trial
Source: PLoS One. 2021 Apr 1;16(4):e0248567. doi: 10.1371/journal.pone.0248567 (PMC8016229; doi:10.1371/journal.pone.0248567)
Supplement: S3 Table — (PDF) [file pone.0248567.s012.pdf]

S3 Table. **Tenth percentile of the average adherence of participants to treatments by subpopulation of the WARFA trial (adherence in percentage).**

Population	Marevan (%)	UQW (%)	TW (%)
**Complete cases**<br>Mean INR and mean warfarin dose per week	86.2<br>(n=56)	86.2<br>(n=55)	86.9<br>(n=55)
**Complete cases**<br>∆ INR, ∆ dose and mean TTR	90.5<br>(n=52)	86.2<br>(n=53)	86.9<br>(n=51)
**First treatment period group**<br>Mean INR and mean warfarin dose per week	90.5<br>(n=25)	81.3<br>(n=29)	75.0<br>(n=30)
**First treatment period group**<br>∆ INR, ∆ dose and mean TTR	95.7<br>(n=22)	81.3<br>(n=29)	93.1<br>(n=28)
**Modified intention-to-treat**<br>Mean INR and mean warfarin dose per week	87.0<br>(n=70)	86.9<br>(n=65)	86.2<br>(n=72)
**Modified intention-to-treat**<br>∆ INR, ∆ dose and mean TTR	90.5<br>(n=65)	86.9<br>(n=62)	86.9<br>(n=66)

∆INR: INR variability; INR: international normalized rate; TW: Teuto warfarin; TTR: time in therapeutic range; UQW: União Química warfarin.
