# Supplementary material for: Examining therapeutic equivalence between branded and generic warfarin in Brazil: The WARFA crossover randomized controlled trial
Source: PLoS One. 2021 Apr 1;16(4):e0248567. doi: 10.1371/journal.pone.0248567 (PMC8016229; doi:10.1371/journal.pone.0248567)
Supplement: S4 Table — (PDF) [file pone.0248567.s013.pdf]

S4 Table. Frequency of clinical events in the WARFA trial by warfarin product.

	Marevan	UQW	TW	Total
**Thromboembolic events**, n	1[a]	0	1[b]	2
**Major bleedings**, n	1[c]	0	1[d]	2
**Minor bleedings**, n	16[e]	21	21	58[f]
**Death**, n	0	0	1	1
**Other**, n	0	0	2[g]	2

TW: Teuto warfarin; UQW: União Química warfarin.

[a] Transient ischemic attack without further consequences on the 4th period of the trial; study treatment was resumed, and patient completed the trial. Patient had already used UQW and TW in the previous periods of the trial.

[b] Ischemic stroke that led to hospitalization on the 4th period of the trial; patient died days later due to pulmonary edema, hypertensive cardiomyopathy, hypertension and diabetes mellitus. Patient had already used Marevan and UQW in the previous periods of the trial.

[c] Gastrointestinal bleeding on the 2nd period of the trial that led to suspension of the anticoagulation for an indefinite period; patient was excluded from further periods of the trial without using UQW or TW, which were assigned for the following study periods.

[d] Intraretinal (vitreous) hemorrhage on the 2nd period of the trial; as the patient had high risk of stroke and the event was benign, study treatments were maintained, and patient completed the trial, using Marevan and UQW in the following study periods.

[e] One gastrointestinal bleeding event, despite being minor, led to suspension of the anticoagulation (by an external physician) for an indefinite period; patient was excluded from further periods of the trial without using UQW or TW, which were assigned for the following study periods.

[f] Registered in 35 patients. The first occurrence of the minor bleeding was in the 1st period of the trial for 13 patients; on the 2nd period for 9 patients, on the 3rd period for 9 patients and on the 4th period for 5 patients.

[g] One patient reported recurrent pruritus only immediately after taking TW, on the 4th period of the trial, what led to discontinuation of that warfarin formulation; patient had used Marevan and UQW without experiencing the same type of type I hypersensitivity reaction. Another patient reported, on the 4th period of the trial, a purple coloration inside his mouth repeatedly only after taking TW, but not after taking UQW or Marevan. Both events had no further consequences.
