# Supplementary material for: Examining therapeutic equivalence between branded and generic warfarin in Brazil: The WARFA crossover randomized controlled trial
Source: PLoS One. 2021 Apr 1;16(4):e0248567. doi: 10.1371/journal.pone.0248567 (PMC8016229; doi:10.1371/journal.pone.0248567)
Supplement: S5 Table — (PDF) [file pone.0248567.s014.pdf]

S5 Table. Baseline characteristics of the subpopulation First treatment period group, by sequence, for the outcomes of mean INR and mean warfarin dose per week.

	Sequence A (n=16) UQW	Sequence B (n=14) M	Sequence C (n=16) M	Sequence D (n=16) TW	Sequence E (n=15) TW	Sequence F (n=17) UQW
**Age (years)**, mean (SD)	63.9 (11.9)	69.4 (10.7)	69.0 (7.6)	68.1 (9.5)	65.3 (8.0)	64.0 (8.8)
**Female**, n (%)	8 (50.0)	4 (28.6)	10 (62.5)	4 (25.0)	4 (26.7)	5 (29.4)
**Atrial Fibrillation**, n (%)	15 (93.7)[a]	13 (92.9)[a]	15 (93.7)	15 (93.7)	15 (100.0)[b]	14 (82.3)
Valvular AF, n (%)	2 (12.5)	0 (0.0)	0 (0.0)	0 (0.0)	0 (0.0)	0 (0.0)
**Atrial Flutter**, n (%)	2 (12.5)[a]	2 (14.3)[a]	1 (6.2)	1 (6.2)	2 (13.3)[b]	3 (17.6)
Valvular AFL, n (%)	0 (0.0)	0 (0.0)	0 (0.0)	0 (0.0)	0 (0.0)	1 (5.9)
**CHA_2DS_2VASc**, mean (SD)	3.3 (1.3)	3.8 (1.9)	3.4 (1.1)	3.4 (1.8)	2.9 (1.1)	3.2 (1.4)
**CHA_2DS_2VASc**, n (%)						
0	0 (0.0)	0 (0.0)	0 (0.0)	0 (0.0)	0 (0.0)	0 (0.0)
1	1 (6.2)	1 (7.1)	0 (0.0)	2 (12.5)	2 (13.3)	1 (5.9)
≥2	15 (93.7)	13 (92.8)	16 (100.0)	14 (87.5)	13 (86.7)	16 (94.1)
**HAS-BLED**, mean (SD)	1.3 (1.3)	1.9 (1.3)	1.2 (0.8)	1.4 (0.9)	1.4 (1.0)	1.2 (0.8)
**HAS-BLED**, n (%)						
0	6 (37.5)	2 (14.3)	3 (18.7)	2 (12.5)	3 (20.0)	3 (17.6)
1-2	7 (43.7)	8 (57.1)	12 (75.0)	12 (75.0)	10 (66.7)	13 (76.5)
≥3	3 (18.7)	4 (28.6)	1 (6.25)	2 (12.5)	2 (13.3)	1 (5.9)
**CHF or LV dysfunction**, n (%)	6 (37.5)	6 (42.9)	3 (18.7)	7 (43.7)	4 (26.7)	6 (35.3)
**Hypertension**, n (%)	15 (93.7)	14 (100.0)	16 (100.0)	16 (100.0)	15 (100.0)	16 (94.1)
**Diabetes mellitus**, n (%)	2 (12.5)	3 (21.4)	3 (18.7)	7 (43.7)	4 (26.7)	5 (29.4)
**Stroke**, n (%)	2 (12.5)	3 (21.4)	2 (12.5)	2 (12.5)	1 (6.7)	1 (5.9)
**TIA**, n (%)	0 (0.0)	0 (0.0)	0 (0.0)	0 (0.0)	1 (6.7)	1 (5.9)
**TE**, n (%)	1 (6.2)	1 (7.1)	1 (6.2)	0 (0.0)	0 (0.0)	1 (5.9)
**MI**, n (%)	3 (18.7)	5 (35.7)	1 (6.2)	3 (18.7)	1 (6.7)	4 (23.5)
**PAD**, n (%)	2 (12.5)	1 (7.1)	1 (6.2)	1 (6.2)	0 (0.0)	3 (17.6)
**INR**, mean (SD)	2.49 (0.81)	2.54 (0.60)	2.50 (0.67)	2.49 (0.79)	2.38 (0.52)	2.53 (0.51)
**Warfarin dose (mg) per week**, mean (SD)	30.8 (14.8)	30.6(11.4)[c]	28.9 (9.2)	36.4 (16.8)	30.2 (12.1)	28.2 (10.8)

AF: atrial fibrillation; AFL: atrial flutter; CHF: congestive heart failure; ∆INR: INR variability; INR: international normalized ratio; LV: left ventricular; M: Marevan; MI: myocardial infarction; PAD: peripheral arthery disease; SD: standard deviation; TW: Teuto warfarin; TE: thromboembolism; TIA: transient ischemic attack; TTR: time in therapeutic range; UQW: União Química warfarin.

[a] 1 patient with both AF and AFL.

[b] 2 patients with both AF and AFL.

[c] n=10. We did not have the baseline weekly dose of one of the patients. He used to take 1 tablet of 5 mg warfarin every other day, i.e., he could have taken either 15 mg or 20 mg in the 7 days prior to the randomization.
