# Supplementary material for: Examining therapeutic equivalence between branded and generic warfarin in Brazil: The WARFA crossover randomized controlled trial
Source: PLoS One. 2021 Apr 1;16(4):e0248567. doi: 10.1371/journal.pone.0248567 (PMC8016229; doi:10.1371/journal.pone.0248567)
Supplement: S6 Table — (PDF) [file pone.0248567.s015.pdf]

S6 Table. Baseline characteristics of the subpopulation First treatment period group, by sequence, for the outcomes of ΔINR, Δ dose, and mean TTR.

	Sequence A (n=16) UQW	Sequence B (n=11) M	Sequence C (n=14) M	Sequence D (n=15) TW	Sequence E (n=13) TW	Sequence F (n=15) UQW
**Age (years)**, mean (SD)	63.9 (11.9)	69.4 (12.1)	69.9 (7.7)	67.7 (9.7)	65.8 (8.3)	64.7 (8.5)
**Female**, n (%)	8 (50.0)	3 (27.3)	9 (64.3)	3 (20.0)	3 (23.1)	4 (26.7)
**Atrial Fibrillation**, n (%)	15 (93.7)[a]	10 (90.9)[a]	13 (92.9)	14 (93.3)	13 (100.0)[b]	13 (86.7)
Valvular AF, n (%)	2 (12.5)	0 (0.0)	0 (0.0)	0 (0.0)	0 (0.0)	0 (0.0)
**Atrial Flutter**, n (%)	2 (12.5)[a]	2 (18.2)[a]	1 (7.1)	1 (6.7)	2 (15.4)[b]	2 (13.3)
Valvular AFL, n (%)	0 (0.0)	0 (0.0)	0 (0.0)	0 (0.0)	0 (0.0)	0 (0.0)
**CHA_2DS_2VASc**, mean (SD)	3.3 (1.3)	3.8 (2.1)	3.3 (1.1)	3.3 (1.7)	2.8 (1.1)	3.3 (1.5)
**CHA_2DS_2VASc**, n (%)						
0	0 (0.0)	0 (0.0)	0 (0.0)	0 (0.0)	0 (0.0)	0 (0.0)
1	1 (6.2)	1 (9.1)	0 (0.0)	2 (13.3)	2 (15.4)	1 (6.7)
≥2	15 (93.7)	10 (90.9)	14 (100.0)	13 (86.7)	11 (84.6)	14 (93.3)
**HAS-BLED**, mean (SD)	1.3 (1.3)	1.4 (0.9)	1.3 (0.8)	1.5 (0.9)	1.4 (1.0)	1.2 (0.9)
**HAS-BLED**, n (%)						
0	6 (37.5)	2 (18.2)	2 (14.3)	2 (13.3)	3 (23.1)	3 (20.0)
1-2	7 (43.7)	8 (72.7)	11 (78.6)	11 (73.3)	8 (61.5)	11 (73.3)
≥3	3 (18.7)	1 (9.1)	1 (7.1)	2 (13.3)	2 (15.4)	1 (6.7)
**CHF or LV dysfunction**, n (%)	6 (37.5)	6 (54.5)	2 (14.3)	6 (40.0)	4 (30.8)	5 (33.3)
**Hypertension**, n (%)	15 (93.7)	11 (100.0)	14 (100.0)	15 (100.0)	13 (100.0)	14 (93.3)
**Diabetes mellitus**, n (%)	2 (12.5)	3 (27.3)	3 (21.4)	6 (40.0)	3 (23.1)	4 (26.7)
**Stroke**, n (%)	2 (12.5)	1 (9.1)	1 (7.1)	2 (13.3)	0 (0.0)	1 (6.7)
**TIA**, n (%)	0 (0.0)	0 (0.0)	0 (0.0)	0 (0.0)	1 (7.7)	1 (6.7)
**TE**, n (%)	1 (6.2)	1 (9.1)	1 (7.1)	0 (0.0)	0 (0.0)	1 (6.7)
**MI**, n (%)	3 (18.7)	4 (36.4)	1 (7.1)	2 (13.3)	1 (7.7)	4 (26.7)
**PAD**, n (%)	2 (12.5)	1 (9.1)	1 (7.1)	1 (6.7)	0 (0.0)	3 (20.0)
**INR**, mean (SD)	2.49 (0.81)	2.47 (0.58)	2.47 (0.70)	2.52 (0.81)	2.36 (0.52)	2.53 (0.53)
**Warfarin dose (mg) per week**, mean (SD)	30.8 (14.8)	28.5(12.3)[c]	28.9 (7.5)	36.7 (17.3)	28.8 (12.6)	28.8 (11.4)

AF: atrial fibrillation; AFL: atrial flutter; CHF: congestive heart failure; ΔINR: INR variability; INR: international normalized ratio; LV: left ventricular; M: Marevan; MI: myocardial infarction; PAD: peripheral arthery disease; SD: standard deviation; TW: Teuto warfarin; TE: thromboembolism; TIA: transient ischemic attack; TTR: time in therapeutic range; UQW: União Química warfarin.

[a] 1 patient with both AF and AFL.

[b] 2 patients with both AF and AFL.

[c] n=10. We did not have the baseline weekly dose of one of the patients. He used to take 1 tablet of 5 mg warfarin every other day, i.e., he could have taken either 15 mg or 20 mg in the 7 days prior to the randomization.
