# Supplementary material for: Examining therapeutic equivalence between branded and generic warfarin in Brazil: The WARFA crossover randomized controlled trial
Source: PLoS One. 2021 Apr 1;16(4):e0248567. doi: 10.1371/journal.pone.0248567 (PMC8016229; doi:10.1371/journal.pone.0248567)
Supplement: S8 Table — (PDF) [file pone.0248567.s017.pdf]

S8 Table. Baseline characteristics, by sequence and period, of the subpopulation Modified intention-to-treat for the outcomes of ∆INR, ∆ dose, and mean TTR.

	Sequence A (n=16)	Sequence B (n=12)	Sequence C (n=14)	Sequence D (n=15)	Sequence E (n=13)	Sequence F (n=15)
**Age (years)**, mean (SD)	63.9 (11.9)	69.7 (11.6)	69.9 (7.7)	67.7 (9.7)	65.8 (8.3)	64.7 (8.5)
**Female**, n (%)	8 (50.0)	4 (33.3)	9 (64.3)	3 (20.0)	3 (23.1)	4 (26.7)
**Atrial Fibrillation**, n (%)	15 (93.7)[a]	11 (91.7)[a]	13 (92.9)	14 (93.3)	13 (100.0)[b]	13 (86.7)
Valvular AF, n (%)	2 (12.5)	0 (0.0)	0 (0.0)	0 (0.0)	0 (0.0)	0 (0.0)
**Atrial Flutter**, n (%)	2 (12.5)[a]	2 (16.7)[a]	1 (7.1)	1 (6.7)	2 (15.4)[b]	2 (13.3)
Valvular AFL, n (%)	0 (0.0)	0 (0.0)	0 (0.0)	0 (0.0)	0 (0.0)	0 (0.0)
**CHA_2DS_2VASc**, mean (SD)	3.3 (1.3)	3.9 (2.0)	3.3 (1.1)	3.3 (1.7)	2.8 (1.1)	3.3 (1.5)
**CHA_2DS_2VASc**, n (%)						
0	0 (0.0)	0 (0.0)	0 (0.0)	0 (0.0)	0 (0.0)	0 (0.0)
1	1 (6.2)	1 (8.3)	0 (0.0)	2 (13.3)	2 (15.4)	1 (6.7)
≥2	15 (93.7)	11 (91.7)	14 (100.0)	13 (86.7)	11 (84.6)	14 (93.3)
**HAS-BLED**, mean (SD)	1.3 (1.3)	1.6 (1.0)	1.3 (0.8)	1.5 (0.9)	1.4 (1.0)	1.2 (0.9)
**HAS-BLED**, n (%)						
0	6 (37.5)	2 (16.7)	2 (14.3)	2 (13.3)	3 (23.1)	3 (20.0)
1-2	7 (43.7)	8 (66.7)	11 (78.6)	11 (73.3)	8 (61.5)	11 (73.3)
≥3	3 (18.7)	2 (16.7)	1 (7.1)	2 (13.3)	2 (15.4)	1 (6.7)
**CHF or LV dysfunction**, n (%)	6 (37.5)	6 (50.0)	2 (14.3)	6 (40.0)	4 (30.8)	5 (33.3)
**Hypertension**, n (%)	15 (93.7)	12 (100.0)	14 (100.0)	15 (100.0)	13 (100.0)	14 (93.3)
**Diabetes mellitus**, n (%)	2 (12.5)	3 (25.0)	3 (21.4)	6 (40.0)	3 (23.1)	4 (26.7)
**Stroke**, n (%)	2 (12.5)	2 (16.7)	1 (7.1)	2 (13.3)	0 (0.0)	1 (6.7)
**TIA**, n (%)	0 (0.0)	0 (0.0)	0 (0.0)	0 (0.0)	1 (7.7)	1 (6.7)
**TE**, n (%)	1 (6.2)	1 (8.3)	1 (7.1)	0 (0.0)	0 (0.0)	1 (6.7)
**MI**, n (%)	3 (18.7)	4 (33.3)	1 (7.1)	2 (13.3)	1 (7.7)	4 (26.7)
**PAD**, n (%)	2 (12.5)	1 (8.3)	1 (7.1)	1 (6.7)	0 (0.0)	3 (20.0)
**Baseline INR in the 1st period**, mean (SD)	2.49 (0.81)	2.47 (0.56)	2.47 (0.70)	2.52 (0.81)	2.36 (0.52)	2.53 (0.53)
**Baseline warfarin dose (mg) per week in the 1st period**, mean (SD)	30.8 (14.8)	29.3(12.0)[c]	28.9 (7.5)	36.7 (17.3)	28.8 (12.6)	28.8 (11.4)
**Baseline INR in the 2nd period** [d], mean (SD)	2.49 (0.75)	2.83 (0.63)	2.52 (0.48)	2.81 (0.48)	2.25 (0.52)	2.50 (0.41)
**Baseline warfarin dose (mg) per week in the 2nd period** [d], mean (SD)	33.7 (15.2)	33.1 (9.0)	27.5 (7.3)	37.5 (18.1)	36.1 (12.5)	30.4 (12.2)
**Baseline INR in the 3rd period**[e], mean (SD)	2.54 (0.84)	3.19 (0.61)	2.73 (0.55)	2.56 (0.33)	2.47 (0.76)	2.70 (0.50)
**Baseline warfarin dose (mg) per week in the 3rd period**[e], mean (SD)	29.6 (12.2)	35.6 (12.8)	26.0 (6.8)	37.2 (19.5)	37.9 (13.7)	32.0 (13.0)
**Baseline INR in the 4th**	2.94 (1.32)	2.08 (0.49)	2.54 (0.46)	3.05 (0.69)	2.37 (0.33)	2.73 (0.75)

**period**[f], mean (SD)						
**Baseline warfarin dose (mg) per week in the 4th period** [g], mean (SD)	31.7 (16.1)	31.0 (16.0)	28.9 (7.5)	38.7 (20.2)	36.2 (14.2)	29.1 (12.8)

AF: atrial fibrillation; AFL: atrial flutter; CHF: congestive heart failure; ∆INR: INR variability; INR: international normalized ratio; LV: left ventricular; M: Marevan; MI: myocardial infarction; PAD: peripheral arthery disease; SD: standard deviation; TW: Teuto warfarin; TE: thromboembolism; TIA: transient ischemic attack; TTR: time in therapeutic range; UQW: União Química warfarin.

[a] 1 patient with both AF and AFL.

[b] 2 patients with both AF and AFL.

[c] n=11. We did not have the baseline weekly dose of one of the patients. He used to take 1 tablet of 5 mg warfarin every other day, i.e., he could have taken either 15 mg or 20 mg in the 7 days prior to the randomization.

[d] Sequence A, n=12. Sequence B, n=4. Sequence C, n=11. Sequence D, n=10. Sequence E, n=7. Sequence F, n=13.

[e] Sequence A, n=12. Sequence B, n=4. Sequence C, n=10. Sequence D, n=9. Sequence E, n=6. Sequence F, n=10.

[f] Sequence A, n=13. Sequence B, n=5. Sequence C, n=8. Sequence D, n=8. Sequence E, n=6. Sequence F, n=11.

[g] Sequence A, n=13. Sequence B, n=5. Sequence C, n=7, with one missing dose for a patient that did not follow the instructions on how to take warfarin in the 12th week of treatment and was not sure how she had taken the medication that week. Sequence D, n=8. Sequence E, n=6. Sequence F, n=11.
