# Supplementary material for: Examining therapeutic equivalence between branded and generic warfarin in Brazil: The WARFA crossover randomized controlled trial
Source: PLoS One. 2021 Apr 1;16(4):e0248567. doi: 10.1371/journal.pone.0248567 (PMC8016229; doi:10.1371/journal.pone.0248567)
Supplement: S9 Table — (PDF) [file pone.0248567.s018.pdf]

S9 Table. Baseline characteristics, by sequence and period, of the subpopulation Complete cases for the outcomes of mean INR and mean warfarin dose per week.

	Sequence A (n=13)	Sequence B (n=5)	Sequence C (n=9)	Sequence D (n=9)	Sequence E (n=6)	Sequence F (n=12)
**Age (years)**, mean (SD)	62.8 (11.6)	71.4 (14.4)	70.0 (9.4)	70.4 (8.3)	63.8 (7.1)	63.9 (8.2)
**Female**, n (%)	8 (61.5)	2 (40.0)	6 (66.7)	1 (11.1)	2 (33.3)	4 (33.3)
**Atrial Fibrillation**, n (%)	12 (92.3)	4 (80.0)	8 (88.9)	9 (100.0)	6 (100.0)	11 (91.7)
Valvular AF, n (%)	2 (15.4)	0 (0.0)	0 (0.0)	0 (0.0)	0 (0.0)	0 (0.0)
**Atrial Flutter**, n (%)	1 (7.9)	1 (20.0)	1 (11.1)	0 (0.0)	0 (0.0)	1 (8.3)
Valvular AFL, n (%)	0 (0.0)	0 (0.0)	0 (0.0)	0 (0.0)	0 (0.0)	0 (0.0)
**CHA_2DS_2VASc**, mean (SD)	3.2 (1.4)	5.0 (2.0)	3.3 (1.1)	4.0 (1.6)	3.0 (1.1)	3.4 (1.6)
**CHA_2DS_2VASc**, n (%)						
0	0 (0.0)	0 (0.0)	0 (0.0)	0 (0.0)	0 (0.0)	0 (0.0)
1	1 (7.7)	0 (0.0)	0 (0.0)	0 (0.0)	1 (16.7)	1 (8.3)
≥2	12 (92.3)	5 (100.0)	9 (100.0)	9 (100.0)	5 (83.3)	11 (91.7)
**HAS-BLED**, mean (SD)	1.2 (1.2)	1.4 (1.1)	1.4 (0.9)	1.7 (1.1)	1.3 (1.2)	1.1 (0.7)
**HAS-BLED**, n (%)						
0	5 (38.5)	1 (20.0)	1 (11.1)	2 (22.2)	2 (33.3)	2 (16.7)
1-2	6 (46.1)	3 (60.0)	7 (77.8)	5 (55.5)	3 (50.0)	10 (83.3)
≥3	2 (15.4)	1 (20.0)	1 (11.1)	2 (22.2)	1 (16.7)	0 (0.0)
**CHF or LV dysfunction**, n (%)	4 (30.8)	4 (80.0)	0 (0.0)	5 (55.6)	2 (33.3)	5 (41.7)
**Hypertension**, n (%)	13 (100.0)	5 (100.0)	9 (100.0)	9 (100.0)	6 (100.0)	11 (91.7)
**Diabetes mellitus**, n (%)	1 (7.7)	2 (40.0)	1 (11.1)	5 (55.6)	2 (33.3)	3 (25.0)
**Stroke**, n (%)	1 (7.7)	1 (20.0)	1 (11.1)	2 (22.2)	0 (0.0)	1 (8.3)
**TIA**, n (%)	0 (0.0)	0 (0.0)	0 (0.0)	0 (0.0)	1 (16.7)	1 (8.3)
**TE**, n (%)	1 (7.7)	1 (20.0)	1 (11.1)	0 (0.0)	0 (0.0)	1 (8.3)
**MI**, n (%)	3 (23.1)	3 (60.0)	0 (0.0)	2 (22.2)	1 (16.7)	3 (25.0)
**PAD**, n (%)	1 (7.7)	1 (20.0)	1 (11.1)	1 (11.1)	0 (0.0)	3 (25.0)
**Baseline INR in the 1st period**, mean (SD)	2.57 (0.83)	2.44 (0.65)	2.41 (0.75)	2.30 (0.70)	2.24 (0.45)	2.45 (0.51)
**Baseline warfarin dose (mg) per week in the 1st period**, mean (SD)	32.3 (15.2)	31.5 (13.5)	29.7 (6.9)	37.8 (18.9)	35.4 (10.9)	29.8 (12.3)
**Baseline INR in the 2nd period**, mean (SD)	2.46 (0.74)	2.67 (0.36)	2.56 (0.51)	2.83 (0.50)	2.30 (0.56)	2.53 (0.41)
**Baseline warfarin dose (mg) per week in the 2nd period**, mean (SD)	32.3 (15.2)	32.5 (13.1)	29.2 (6.6)	37.5 (19.2)	37.5 (13.0)	30.4 (12.8)
**Baseline INR in the 3rd period**, mean (SD)	2.54 (0.80)	3.17 (0.53)	2.77 (0.64)	2.56 (0.33)	2.47 (0.76)	2.72 (0.51)
**Baseline warfarin dose (mg) per week in the 3rd period**, mean (SD)	32.5 (15.7)	32.5 (13.1)	28.0 (7.5)	37.2 (19.5)	37.9 (13.7)	30.6 (12.8)
**Baseline INR in the 4th**	2.94 (1.32)	2.08 (0.49)	2.69 (0.62)	2.98 (0.67)	2.36 (0.33)	2.73 (0.71)

**period**, mean (SD)						
**Baseline warfarin dose (mg) per week in the 4th period**, mean (SD)	31.7 (16.1)	31.0 (16.0)	28.4 (7.1) [a]	37.2 (19.5)	38.3 (13.6)	29.2 (12.2)

AF: atrial fibrillation; AFL: atrial flutter; CHF: congestive heart failure; ΔINR: INR variability; INR: international normalized ratio; LV: left ventricular; M: Marevan; MI: myocardial infarction; PAD: peripheral arthery disease; SD: standard deviation; TW: Teuto warfarin; TE: thromboembolism; TIA: transient ischemic attack; TTR: time in therapeutic range; UQW: União Química warfarin.

[a] n=8, with one missing dose for a patient that did not follow the instructions on how to take warfarin in the 12th week of treatment and was not sure how she had taken the medication that week.
