# Supplementary material for: Examining therapeutic equivalence between branded and generic warfarin in Brazil: The WARFA crossover randomized controlled trial
Source: PLoS One. 2021 Apr 1;16(4):e0248567. doi: 10.1371/journal.pone.0248567 (PMC8016229; doi:10.1371/journal.pone.0248567)
Supplement: S10 Table — (PDF) [file pone.0248567.s019.pdf]

S10 Table. Baseline characteristics, by sequence and period, of the subpopulation Complete cases for the outcomes of ∆INR, ∆ dose, and mean TTR.

	Sequence A (n=10)	Sequence B (n=2)	Sequence C (n=7)	Sequence D (n=8)	Sequence E (n=5)	Sequence F (n=9)
**Age (years)**, mean (SD)	60.4 (12.1)	73.0 (24.0)	70.3 (10.8)	69.4 (8.2)	63.8 (7.9)	64.7 (7.4)
**Female**, n (%)	7 (70.0)	0 (0.0)	4 (57.1)	1 (12.5)	2 (40.0)	2 (22.2)
**Atrial Fibrillation**, n (%)	9 (90.0)	2 (100.0)	6 (85.7)	8 (100.0)	5 (100.0)	8 (88.9)
Valvular AF, n (%)	1 (10.0)	0 (0.0)	0 (0.0)	0 (0.0)	0 (0.0)	0 (0.0)
**Atrial Flutter**, n (%)	1 (10.0)	0 (0.0)	1 (14.3)	0 (0.0)	0 (0.0)	1 (11.1)
Valvular AFL, n (%)	0 (0.0)	0 (0.0)	0 (0.0)	0 (0.0)	0 (0.0)	0 (0.0)
**CHA_2DS_2VASc**, mean (SD)	3.0 (1.2)	4.0 (2.8)	3.4 (1.3)	3.9 (1.7)	3.0 (1.2)	3.3 (1.5)
**CHA_2DS_2VASc**, n (%)						
0	0 (0.0)	0 (0.0)	0 (0.0)	0 (0.0)	0 (0.0)	0 (0.0)
1	1 (10.0)	0 (0.0)	0 (0.0)	0 (0.0)	1 (20.0)	1 (11.1)
≥2	9 (90.0)	2 (100.0)	7 (100.0)	8 (100.0)	4 (80.0)	8 (88.9)
**HAS-BLED**, mean (SD)	1.0 (1.2)	0.5 (0.7)	1.3 (0.9)	1.6 (1.2)	1.6 (1.1)	1.2 (0.7)
**HAS-BLED**, n (%)						
0	5 (50.0)	1 (50.0)	1 (14.3)	2 (25.0)	1 (20.0)	1 (11.1)
1-2	3 (30.0)	1 (50.0)	5 (71.4)	4 (50.0)	3 (60.0)	8 (89.8)
≥3	2 (20.0)	0 (0.0)	1 (14.3)	2 (25.0)	1 (20.0)	0 (0.0)
**CHF or LV dysfunction**, n (%)	4 (40.0)	1 (50.0)	0 (0.0)	4 (50.0)	2 (40.0)	3 (33.3)
**Hypertension**, n (%)	10 (100.0)	2 (100.0)	7 (100.0)	8 (100.0)	5 (100.0)	8 (88.9)
**Diabetes mellitus**, n (%)	0 (0.0)	1 (50.0)	1 (14.3)	5 (62.5)	2 (40.0)	3 (33.3)
**Stroke**, n (%)	0 (0.0)	0 (0.0)	1 (14.3)	2 (25.0)	0 (0.0)	1 (11.1)
**TIA**, n (%)	0 (0.0)	0 (0.0)	0 (0.0)	0 (0.0)	0 (0.0)	1 (11.1)
**TE**, n (%)	1 (10.0)	0 (0.0)	1 (14.3)	0 (0.0)	0 (0.0)	0 (0.0)
**MI**, n (%)	2 (20.0)	2 (100.0)	0 (0.0)	1 (12.5)	1 (20.0)	3 (33.3)
**PAD**, n (%)	1 (10.0)	0 (0.0)	1 (14.3)	1 (12.5)	0 (0.0)	2 (22.2)
**Baseline INR in the 1st period**, mean (SD)	2.62 (0.93)	2.13 (0.36)	2.32 (0.65)	2.34 (0.74)	2.19 (0.48)	2.37 (0.46)
**Baseline warfarin dose (mg) per week in the 1st period**, mean (SD)	30.2 (12.6)	37.5 (3.5)	28.6 (5.6)	39.4 (19.5)	35.0 (12.1)	31.1 (13.3)
**Baseline INR in the 2nd period**, mean (SD)	2.62 (0.74)	2.63 (0.30)	2.49 (0.56)	2.73 (0.42)	2.41 (0.54)	2.54 (0.39)
**Baseline warfarin dose (mg) per week in the 2nd period**, mean (SD)	30.2 (12.6)	38.7 (1.8)	27.8 (4.9)	39.1 (19.9)	37.5 (14.6)	31.9 (13.8)
**Baseline INR in the 3rd period**, mean (SD)	2.61 (0.90)	2.99 (0.91)	2.71 (0.63)	2.58 (0.35)	2.63 (0.73)	2.64 (0.49)
**Baseline warfarin dose (mg) per week in the 3rd period**, mean (SD)	30.0 (12.6)	38.7 (1.8)	26.4 (5.9)	38.7 (20.2)	38.0 (15.3)	32.2 (13.8)
**Baseline INR in the 4th period**, mean (SD)	3.25 (1.36)	2.40 (0.35)	2.55 (0.49)	3.05 (0.69)	2.36 (0.36)	2.81 (0.76)

**Baseline warfarin dose (mg) per week in the 4th period**, mean (SD)	29.2 (13.1)	37.5 (3.5)	27.1 (6.2) [a]	38.7 (20.2)	38.0 (15.1)	30.3 (13.3)

AF: atrial fibrillation; AFL: atrial flutter; CHF: congestive heart failure; ∆INR: INR variability; INR: international normalized ratio; LV: left ventricular; M: Marevan; MI: myocardial infarction; PAD: peripheral arthery disease; SD: standard deviation; TW: Teuto warfarin; TE: thromboembolism; TIA: transient ischemic attack; TTR: time in therapeutic range; UQW: União Química warfarin.

[a] n=6, with one missing dose for a patient that did not follow the instructions on how to take warfarin in the 12th week of treatment and was not sure how she had taken the medication that week.
