# Supplementary material for: Examining therapeutic equivalence between branded and generic warfarin in Brazil: The WARFA crossover randomized controlled trial
Source: PLoS One. 2021 Apr 1;16(4):e0248567. doi: 10.1371/journal.pone.0248567 (PMC8016229; doi:10.1371/journal.pone.0248567)
Supplement: S1 Appendix — (PDF) [file pone.0248567.s020.pdf]

S1 Appendix. Protocol for adjustment of the warfarin dose applied in the WARFA trial.

INR	Suggested adjustment of the warfarin dose
< 1.5	Increase weekly dose by 10% to 20%<br>Consider giving an extra dose<br>Retest INR in 4 to 8 days or per Investigator discretion
1.5 to < 2	Increase weekly dose by 5% to 10%<br>Retest INR in 7 to 14 days or per Investigator discretion
2.0 to 3.0	No change
> 3.0 to 3.5	Decrease weekly dose by 0% to 20%<br>Retest INR per Investigator discretion
> 3.5 to 4.0	Withhold 0 to 1 dose<br>and/or<br>Decrease weekly dose by 0% to 20%<br>Retest INR per Investigator discretion
> 4.0 but < 5.0	Withhold 1 to 2 doses<br>and<br>Decrease weekly dose by 0% to 20%<br>and<br>Retest INR in 3 to 7 days or per Investigator discretion
5.0 to < 9.0<br>without<br>significant<br>bleeding	Withhold 1 to 2 doses<br>Retest INR in 1 to 2 days or per Investigator discretion<br>Resume dosing once INR < 3.0, but with weekly dose decreased by 5% to 20%<br>If the subject needs urgent surgery, then the subject should receive Fresh Frozen Plasma
> 9.0<br>without<br>significant<br>bleeding	Withhold study drug<br>Give Vitamin K (single 2.5 to 5 mg oral dose)<br>Repeat INR test daily until INR < 5.0<br>If INR remains too high, more Vitamin K doses can be considered.<br>Resume dosing once INR < 3.0, but with weekly dose decreased by 10% to 20%.<br>If the subject needs urgent surgery, then the subject should receive Fresh Frozen Plasma

Adapted from the protocol for the ENGAGE AF-TIMI 48 trial[1].

References:

1. Giugliano RP, Ruff CT, Braunwald E, Murphy SA, Wiviott SD, Halperin JL, et al. Edoxaban versus Warfarin in patients with Atrial fibrillation. N Engl J Med. 2013;369(22):2093–104.
