# Supplementary material for: Examining therapeutic equivalence between branded and generic warfarin in Brazil: The WARFA crossover randomized controlled trial
Source: PLoS One. 2021 Apr 1;16(4):e0248567. doi: 10.1371/journal.pone.0248567 (PMC8016229; doi:10.1371/journal.pone.0248567)
Supplement: S2 Appendix — (PDF) [file pone.0248567.s021.pdf]

S2 Appendix. Changes to the study protocol.

Item in the original protocol or statistical analysis plan	Change applied	Timing	Reason
Inclusion of subjects with **nonvalvular** atrial fibrillation (AF)	Inclusion of patients with AF **and/or** atrial flutter (AFL) **without mechanical prosthetic valves**	• After study start<br>• Before the statistical analysis plan stage	Due to the difficulty of recruiting participants, we broadened our inclusion criteria. Patients with any of these diagnosis are anticoagulated with warfarin aiming the same goal INR, so we did not expect this would bias our mean INR nor other outcomes.
Keeping in the trial only patients that reach a **time in therapeutic range** (TTR) **of at least 70%** in the first period of the trial	Keeping in the trial only patients that:<br>• have at least one of the three INR results from the first period **within the therapeutic range** (between 2.0 and 3.0), and<br>• have only a small difference (**equal to or smaller than 0.8) in their INR results** collected at weeks three and four of the study	• After study start<br>• Before the statistical analysis plan stage	We planned the TTR criteria to select patients with consistent INR results, what would allow an easier identification of differential effects of the warfarins. However, this criteria could also have led us to exclude patients of the utmost interest to us, i.e., patients that had their INRs pulled out of the therapeutic range due to the use of a different warfarin in the trial. Therefore, we adopted the criteria of a small difference in the consecutive INR results collected in the first period of the trial, i.e. even if the INRs end up outside the therapeutic range, the requirement to continue in the trial would be stability of INR results. We also added the requirement of at least one of the INRs within the therapeutic range to ensure patient safety. This was done to prevent patients with difficult INR control to proceed in the trial thus being submitted to potentially deleterious increased INR instability that could be caused by the switch of warfarins within the study.
Mean INR as a **primary outcome**	∆INR as a **primary outcome** and mean INR as a **secondary outcome**	• After study start<br>• At the statistical analysis plan stage<br>• Before data analysis	We considered that being an average between only two values, the mean INR would not be sensitive enough to detect important variations in the warfarins' effects. We then included the ∆INR to address this concern, and the mean INR was changed to a secondary outcome. These outcomes address slightly different concerns. The ΔINR is intended to address variation in the INR results, i.e. warfarins that result in inconsistent INR results, while the mean INRs is more focused to address systematically lower or higher mean INR results, i.e. generic warfarins that, despite being bioequivalent to the brand medication have very different bioavailabilities when compared to each other.

Item in the original protocol or statistical analysis plan	Change applied	Timing	Reason
Mean prothrombin time (PT) as a **secondary outcome**	Mean prothrombin time (PT) **not analyzed**	• After study start<br>• At the statistical analysis plan stage<br>• Before data analysis	We considered that the PT would not add further information to our results, given that the mean INR, more widespread used nowadays, was already within our outcomes and it is calculated from the PT.
Not initially planned.	Δdose as a **secondary outcome**	• After study start<br>• At the statistical analysis plan stage<br>• Before data analysis	We decided including this outcome because we considered that it would be more sensitive than the mean warfarin dose to detect important variations in the warfarins' effects.
The dichotomous outcomes (**TTR**, thromboembolic events, bleeding) will be **analyzed by McNemar's test**	**TTR** analyzed as continuous outcome using **multilevel mixed-effects linear regression with random intercepts**	• After study start<br>• At the statistical analysis plan stage<br>• Before data analysis	Considering that the TTR is a continuous outcome, we decided to analyze it as is.
First period of the trial **shorter** than the others, lasting 21 instead of 28 days	First period of the trial with the **same duration** as the others, i.e., 28 days	• After study start<br>• Before the statistical analysis plan stage	All periods had the same 28-days duration, in order to simplify the study design and allow for two INR measurements, at or after 3 weeks of treatment, in each period (to avoid the carryover effect).
Randomization done by a colleague researcher with a **random number table**. The **researcher** would be **responsible for creating the random sequence and concealing allocation**, besides **blinding** the healthcare providers and the principal investigator (PI)	Randomization and concealment of allocation done by **numbered, opaque and sealed envelopes prepared by the PI**. **Blinding** ensured **by two colleague researchers** that prepared the medication	• Before study start	The original randomization method would require the PI to contact other colleague researcher to get the allocation information for each patient. However, because the colleague was not part of the outpatient clinic team where the study was conducted, the contact would require either mobile phone or internet communication and the prompt availability of the professional, factors that could potentially delay patients' allocation. Therefore, we opted for the randomization by envelopes instead, due to its easy operation, which also facilitated the application of block randomization, and its stand-alone feature, which did not require use of the communication network.

Item in the original protocol or statistical analysis plan	Change applied	Timing	Reason
Blinding patients	Patients **not blinded**	• Before study start	The warfarin tablets from different manufacturers were not identical to each other, neither their packaging. We decided to keep them in their original packaging to prevent any unintended alteration of their stability or bioavailability that could compromise the study results.
At least **48 patients** (**8 in each group**) are needed in order to identify a clinically significant difference of 0.49 in the INR, considering a mean INR of 2.45 with **standard deviation of 0.29**, assuming α = 5% and β = 10%. We plan to **recruit 60 individuals** (10 in each group) to compensate for possible dropouts or exclusions of patients	Our sample size is estimated as **33 patients** (**11 in each group**) in order to identify a clinically significant difference of 0.49 in the INR, considering a mean INR of 2.45 with **standard deviation of 0.34**, assuming α = 5% and β = 10%. Due to the high rate of subjects not meeting the INR stability criteria in the first period we aimed to **recruit 100 patients**	• After study start<br>• Before the statistical analysis plan stage	Although we continued using the same formula, the same significance level and power, we changed the standard deviation for a larger one, in order to have a worst-case scenario estimate. Also, in the original protocol, we mistakenly multiplied the sample size of each group by the number of sequences in the trial (six), instead of the number of treatments in the trial (three).
**Paired-samples t-tests** will be used for the outcome of **mean INR and PT**. The dichotomous outcomes (**TTR, thromboembolic events, bleeding**) will be analyzed by **McNemar's test**	We only analyzed **continuous outcomes** using **multilevel mixed-effects linear regression with random intercepts**	• After study start<br>• At the statistical analysis plan stage<br>• Before data analysis	This decision was made because the original tests are applicable only to comparisons between two groups. Considering that this trial compared three treatments, multiple tests would have to be performed for each outcome, interfering in the overall significance level of the findings.
The dichotomous variables will be **analyzed by intention to treat**, considering the **missing data as an unfavorable outcome**, in which case a sensitivity analysis will be carried out to assess the robustness of the result	We only presented the frequencies of clinical events (thromboembolic, bleeding events, deaths and other adverse effects) and **did not apply hypothesis tests or calculate confidence intervals**	• After study start<br>• At the statistical analysis plan stage<br>• Before data analysis	We have observed very few of these events, as expected, given the limited size of the trial. Thus, we did not conduct hypothesis tests or calculate confidence intervals because we considered we were very likely underpowered for detecting differences between treatments and thus these would not add meaningful information.

Item in the original protocol or statistical analysis plan	Change applied	Timing	Reason
Not initially planned	**We analyzed three populations of subjects**: the Complete cases, the First treatment period group and the Modified intention to treat (ITT)	• After study start<br>• At the statistical analysis plan stage<br>• Before data analysis	Considering that patients were excluded after the first period of the trial on the basis of their INR results, we decided to analyze three populations with different patterns of missing data to assess the impact of these exclusions in the results. Generally, the First treatment period group, which includes data from all randomized patients (but only from the first period of the trial), can be understood as an effectiveness analysis. The complete cases, overall, includes patients that completed the trial, following the study protocol, and thus can be considered an efficacy analysis. The Modified intention to treat population basically included all valid data available. We analyzed the three populations and planned that in case of disagreement within the results of the different analytic populations we would report as the main results the most clinically important findings, i.e., non-equivalence.
We will **not use baseline measures as covariates** since we did not take patients out of the drug (washout) and thus baseline measures are the result of different warfarin formulations that patients were taking before the trial	Outcomes from the First treatment period group population that did not have repeated measures were analyzed with multiple linear regression models. We performed these analyses with **baseline values as covariates** and their results are presented when supporting conclusions that are different from the unadjusted analyses	• After study start<br>• After the statistical analysis plan stage<br>• At the data analysis stage	Regardless of the warfarin used, generally the INR and the warfarin dose of a patient will be highly dependent, respectively, on their most recent INR and warfarin dose. By extension, we considered that the ∆INR, TTR, and ∆dose would also be dependent on these same baseline factors and thus decided for the adjustment.
Not initially planned	We **compared our results** with those calculated by the bootstrap method (with 100 replicates) as an **exploratory sensitivity analysis**	• After study start<br>• After the statistical analysis plan stage<br>• At the data analysis stage	Bootstrapping is a simulation procedure that may be used to assess the uncertainty of sample estimates. We applied it due to concerns regarding the validity of the linear regression models used (the residuals did not fit into a normal distribution).

AF = atrial fibrillation. AFL = atrial flutter. INR = international normalized ratio. ITT = intention to treat. PI = principal investigator. PT = prothrombin time. TTR = time in therapeutic range.
