# Supplementary material for: Examining therapeutic equivalence between branded and generic warfarin in Brazil: The WARFA crossover randomized controlled trial
Source: PLoS One. 2021 Apr 1;16(4):e0248567. doi: 10.1371/journal.pone.0248567 (PMC8016229; doi:10.1371/journal.pone.0248567)
Supplement: S3 Appendix — (PDF) [file pone.0248567.s022.pdf]

S3 Appendix. Definitions used in the WARFA trial.

Endpoint	Definition
**Thromboembolic events**	
Systemic thromboembolic event	Thromboembolic event located on the viscera or extremities diagnosed by acute symptoms followed by relevant diagnostic tests.[1]
Ischemic stroke	Focal neurological deficit of sudden onset as diagnosed by a neurologist, lasting 24h or more and caused by ischemia.[2] Tomography should be used to identify ischemic strokes apart from hemorrhagic strokes.
Transitory ischemic attack	Focal neurological deficit of sudden onset as diagnosed by a neurologist, lasting less than 24h.[2]
**Bleedings**	
Major bleedings	Intracranial hemorrhage, fatal hemorrhage, bleeding that resulted in decrease equal to or greater than 2.0 g/L of serum hemoglobin, bleeding requiring transfusion or bleeding in sensitive areas such as the retina or pericardium.
Minor bleedings	All bleedings apart from major bleedings.
**Other adverse events**	Adverse events that have occurred consistently, immediately after administration of the warfarin formulation.
**Adherence**	Measurement of the percentage of tablets consumed by tablets expected to have been used in the period. Tablets consumed calculated as the difference in tablets dispensed and unused tablets returned. Using this definition, adherence superior to 100% cannot differentiate between warfarin overdose or tablets not returned by the patient to the researcher for other reasons (e.g. patient forgot to return tablets).
**Valid INR value**	INR values were valid if the due measurement was taken when the patient was using the drug to which he/she was assigned without interference of current diarrhoea. INRs were invalidated if collected: 1) when a new drug with major or moderate interactions was used in the last 24 h along with warfarin (for drugs with moderate interactions, they also had

to be used on a continuous basis or at least more than once in the last 24 h); 2) after suspension of a drug with moderate or major interactions previously taken since baseline. We only considered interactions that, according to Micromedex 2.0[3], might alter the INR, and/or the warfarin dose, and/or the risk of bleeding.

References:

1. Petersen P, Boysen G, Godtfredsen J, Andersen ED, Andersen B. Placebo-controlled, randomised trial of warfarin and aspirin for prevention of thromboembolic complications in chronic atrial fibrillation. The Copenhagen AFASAK study. Lancet. 1989;1(8631):175-9.
2. Lip GY, Nieuwlaat R, Pisters R, Lane DA, Crijns HJ. Refining clinical risk stratification for predicting stroke and thromboembolism in atrial fibrillation using a novel risk factor-based approach: the euro heart survey on atrial fibrillation. Chest. 2010;137(2):263-72.
3. Micromedex 2.0. Truven Health Analytics; Greenwood Village. 2017. http:// www.micromedexsolutions.com. Acessed 23 Mar 2017.
