# Supplementary material for: Examining therapeutic equivalence between branded and generic warfarin in Brazil: The WARFA crossover randomized controlled trial
Source: PLoS One. 2021 Apr 1;16(4):e0248567. doi: 10.1371/journal.pone.0248567 (PMC8016229; doi:10.1371/journal.pone.0248567)
Supplement: S1 Protocol — (PDF) [file pone.0248567.s023.pdf]

[Translation of the original protocol approved by the Ethics Committee] Therapeutic equivalence between generic and branded warfarin sodium in adult patients with atrial fibrillation in Brazil - randomized crossover clinical trial

Principal Investigator (PI): Carolina Gomes Freitas

Supervisor: Prof. Dr. Álvaro Nagib Atallah

Co-supervisor: Dr. Dirceu Raposo de Mello

LIST OF ABBREVIATIONS

AFib	Atrial Fibrillation
FDA	Food and Drug Administration
INR	International Normalized Ratio
PI	Principal Investigator
PT	Prothrombin Time
TTR	Time in Therapeutic Range
UNIFESP	Federal University of São Paulo
UTN	Universal Trial Number

1. Background

1.1 Generic drugs

In this introduction, we started describing that generic drugs represent a significant share of the drug market in Brazil and are completely incorporated in the country's health policies (1-4). We mentioned, however, that when it comes to the group of narrow therapeutic index drugs, which includes warfarin, even healthcare providers are suspicious of the equivalency of generic drugs to their branded counterparts (5-7,17-18). Next, we explained the technical requirement that generic drugs must fulfill in order to be considered equivalent to the brand medication, mentioning that some countries, but not Brazil, apply additional requirements to narrow therapeutic index drugs (7-12). Afterwards, we presented some studies that compared the effects of generic and branded warfarins. Randomized clinical trials did not show clinically meaningful differences between generic and branded warfarins, whereas observational studies brought more conflicting results. Nevertheless, none of these studies have used the generic and branded warfarins available in Brazil, limiting their applicability in our country (6, 13-16,19-21). Subsequently, we mentioned an issue that might arise when there is more than one generic drug available for a given brand medication: generics are devised to be equivalent to the brand medication, but not necessarily equivalent to each other (22). Finally, we presented the objectives of this project: run a randomized crossover clinical trial to assess whether the substitution of 1) branded warfarin by any of the generic formulations available in Brazil, or 2) substitution within the generic warfarins, is effective and safe for the patient with atrial fibrillation (AFib). We also added that patients with AFib diagnosis were selected because this was the most common diagnosis among the patients of anticoagulation clinics (23).

1.2 Atrial fibrillation

In this section, we defined AFib and presented its prevalence, the characteristics of these patients and the consequences the disease may incur (24-29). We them presented the role that oral anticoagulation has in the treatment of patients with AFib and presented the drugs available for that treatment (30, 31). We then conclude that despite the benefits of the direct oral anticoagulants (32), warfarin is still the cheapest oral anticoagulant and will probably continue in use for many years.

1.3 Warfarin

In this section, we described warfarin's characteristics: its mechanism of action, pharmacokinetics, the danger of use during pregnancy, side effects, mechanism of drug interactions, how to titrate the optimal dose and the importance of good management of the optimal dose (27, 32-35).

2. Objectives

2.1 Overall objective

Determine, through the mean international normalized ratio (INR) of patients, whether there is therapeutic equivalence between brand and generic warfarin sodium tablets in patients with atrial fibrillation in Brazil.

2.2 Specific objectives

Assess the effects of the different warfarin tablets on:

- the mean INR of patients;
- the mean prothrombin time (PT);
- the proportion of thromboembolic events;
- the proportion of bleedings;
- the time in therapeutic range (TTR);
- adherence to treatment.

3. Hypothesis

The comparison of the mean INR between branded and generic warfarins is within the limit of 0.49 (two-sided, with a significance level of 5%) demonstrating therapeutic equivalence between them.

4. Methods

4.1 Type of study

Randomized crossover controlled trial containing six treatment sequences and divided into four phases with different durations (Figure 1). The study is registered in www.clinicaltrials.gov under the identification NCT02017197 and also has the Universal Trial Number (UTN) U1111-1155-4833.

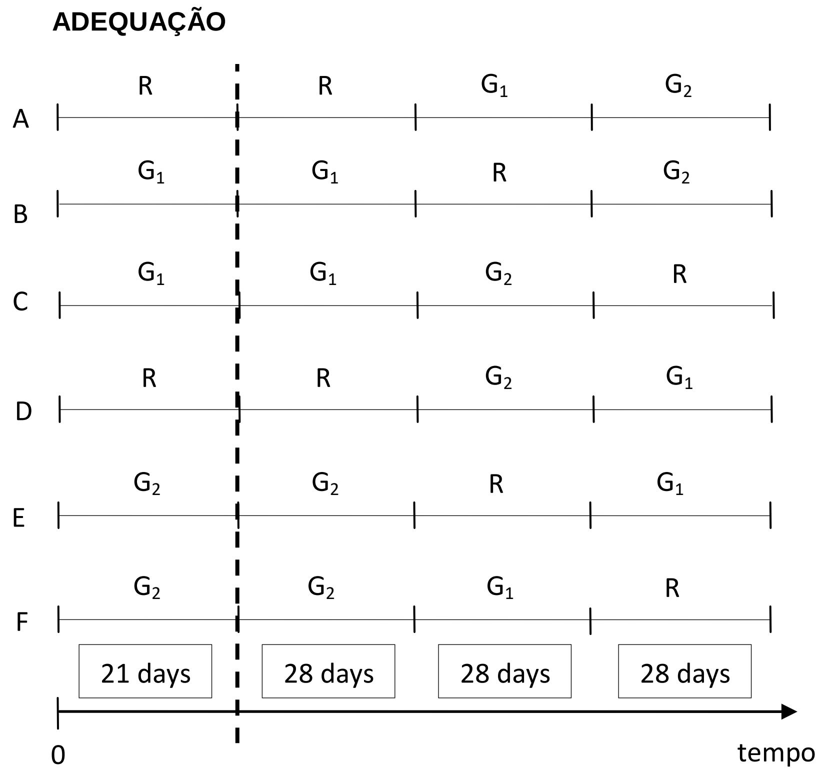

Figure 1: Sequences of treatment for patients randomly allocated to groups A, B, C, D, E or F in relation to time. R: Branded warfarin. G1 and G2: generic warfarin from different manufacturers.

4.2 Setting

Outpatient clinic of the Discipline of Cardiology, Department of Medicine, at the Federal University of São Paulo.

4.3 Participants

4.3.1 Inclusion criteria

Patients who, according to a structured interview, or by review of their medical records, simultaneously meet all the following criteria will be included in the study:

- have a diagnosis of nonvalvular atrial fibrillation (documented by electrocardiogram and echocardiogram) (26);
- have a CHA_2DS_2VASc score greater than or equal to one (for whom is recommended anticoagulation with warfarin);
- are using warfarin;

- are over 18 years of age;
- and agree to participate in the study by signing the Informed Consent Form (Annex I)

4.3.2 Exclusion criteria

Patients who meet any of the following criteria will not be accepted:

- patients to whom the use of anticoagulants is contraindicated (patients using other anticoagulants, or with active bleeding, or with hypersensitivity to warfarin, or with ulcers);
- women of childbearing age, or who are pregnant or breastfeeding;
- patients with thrombocytopenia;
- patients with liver or kidney failure;
- patients with a history of bleeding episodes due to congenital deficiency of coagulation factors;
- patients enrolled in other clinical trials;
- patients not reaching a TTR of at least 70% in the first phase of the study (will be considered non-adherent to therapy);
- patients starting treatment with drugs that are contraindicated or that incur in moderate or major interactions when used concomitantly with warfarin, according to the online database Micromedex 2.0 (37) (Annex II).

4.4 Procedures

Patients will be randomly allocated to one of the treatment sequences (A, B, C, D, E or F, Figure 1) and will start treatment with either the branded warfarin sodium or with one of the two generic versions available in the Brazilian market in August 2013. The first phase of the study will last 21 days. During this period, the patient, who had already been using warfarin, will start the randomly allocated treatment that will also be used in the second phase of the clinical trial. The use of the same treatment in phases one and two aims to avoid the carry-over effect without the interruption of anticoagulation treatment, which would be ethically unacceptable. The duration of three weeks for this phase is equivalent to about seven warfarin half-lives (38), the washout time for elimination of this drug. The duration of this phase will allow us to ensure that the formulation of warfarin used previously does not interfere with the results of the trial.

The second, third and fourth phases of the study will last 28 days each, in order to allow more weekly measurements to assess the effect of therapy with each of the formulations. In the second phase the patients will continue with the treatment started in phase one, and in the third and fourth phases will occur the change of warfarins according to the predetermined sequences for each group.

4.4.1 Randomization

The randomization sequence will be generated by an independent investigator (TFCP) using a random number table created with the Microsoft Excel software. The independent investigator (TFCP) will be responsible for maintaining the confidentiality of the randomization sequence until the data analysis phase, assigning to the medication the initials of the patient and an alphanumeric code that will correlate with the formulation of the warfarin used. The medication will then be dispensed by the principal investigator (CGF) in opaque and identical sealed packages.

4.4.2 Intervention

Patients will receive, according to the study phase, the branded warfarin sodium (Marevan, manufactured by Farmoquímica, Brazil) or one of the generic drugs (one manufactured by União Química Farmacêutica Nacional S/A and the other manufactured by Laboratório Teuto Brasileiro S/A, Brazil) available in the Brazilian market in August 2013.

The prescription of the drug will be issued in the generic drug name, thus allowing the patient to receive both the branded and the generic medications. The tablets available on the market have a similar appearance to each other and the dispensation will be carried out by the PI in the outpatient clinic. Opaque and identical sealed packages for the medication will prevent patients, doctors and the lead investigator from being aware of the warfarin that the patient will use. Patients will receive enough medication to be used in the period between laboratory tests. Afterwards, the returned tablets will be counted with two objectives: to assess and encourage treatment adherence. The dose adjustment will be carried out monthly, or more frequently at medical discretion, if the INR results fall out of the reference range, whose target is maintenance between 2.0 and 3.0.

4.5 Variables and outcomes

Outcome data will be collected by the principal investigator (CGF). Laboratory tests (INR and TP) and clinical outcomes (thromboembolic events and bleeding) will be assessed twice a month; treatment adherence will be assessed monthly.

4.5.1 Primary outcome

- Mean INR

  The average INR of each patient will be compared when using the different warfarin formulations.

4.5.2 Secondary outcomes

- Mean PT

  The mean PT will be evaluated in the same way as the INR.
- Time within the therapeutic range (TTR).

  This ratio is calculated for each patient by the percentage of person-time within the therapeutic range of 2.0 and 3.0 divided by the total person-time contribution by that same individual (39).
- Frequency of thromboembolic events

  It includes ischemic stroke (differentiated from hemorrhagic stroke by tomography) and thromboembolism on the viscera or extremities (diagnosed by acute symptoms and relevant diagnostic tests) (40).
- Frequency of bleedings

  Bleeding events will be classified as major or minor bleedings:
  - Major bleeding: intracranial bleeding, fatal bleeding, blood loss resulting in a decrease greater than or equal to 2.0 g / L of serum hemoglobin, bleeding requiring transfusion, bleeding in sensitive areas such as the retina or pericardium.
  - Minor bleeding: all other bleeding (33).
- Adherence to treatment

  Adherence will be calculated by the amount of tablets returned by the patient.

4.6 Statistical Method

4.6.1 Sample size

At least 48 patients (8 in each group) will be needed in order to identify a clinically significant difference of 0.49 in the INR, considering a mean INR of 2.45 with standard deviation of 0.29, data reported for a branded warfarin in a published study (13). The sample was calculated based on the mean INR outcome, using the statistical method for quantitative outcomes (41) assuming $\alpha = 5\%$ and $\beta = 10\%$, which result in a power of 90%. We plan to recruit 60 individuals (10 in each group) to compensate for possible dropouts or exclusions of patients.

4.6.2 Statistical analysis

All statistical analyzes will have a significance level of 5%. Paired-samples t-tests (also called dependent T-tests) will be used for the outcome of mean INR and PT. Analysis will be performed by protocol for these outcomes i.e., data from patients who start treatment with any drugs that interact with warfarin as previously defined (list in Annex II) will be excluded from the final analysis.

The dichotomous outcomes (TTR, thromboembolic events, bleeding) will be analyzed by McNemar's test. Exploratory analyzes for patient subgroups are not planned. The dichotomous variables will be analyzed by intention to treat, considering the missing data as an unfavorable outcome, in which case a sensitivity analysis will be carried out to assess the robustness of the result.

5. References

1. Brasil. Ministério da Saúde. Política Nacional de Medicamentos. Portaria Nº 3.916, de 30 de outubro de 1998. Brasília: Diário Oficial da União; 1998.

2. Brasil. Ministério da Saúde. Agência Nacional de Vigilância Sanitária. Medicamento Genérico. Brasília.

3. PróGenéricos. Associação Brasileira das Indústrias de Medicamentos Genéricos. Vendas de genérico chegam a 6,3 bi 2013 [updated 26 Julho 2013; cited 2013 19 Agosto]. Available from: http://www.progenericos.org.br/index.php/noticias/328-vendas-de-generico-chegam-a-r-63-bi.

4. PróGenéricos. Associação Brasileira das Indústrias de Medicamentos Genéricos. Mercado [cited 2013 19 Agosto]. Available from: http://www.progenericos.org.br/index.php/mercado.

5. Davit BM, Nwakama PE, Buehler GJ, Conner DP, Haidar SH, Patel DT, et al. Comparing generic and innovator drugs: a review of 12 years of bioequivalence data from the United States Food and Drug Administration. The Annals of pharmacotherapy. 2009 Oct;43(10):1583-97. PubMed PMID: 19776300. Epub 2009/09/25. eng.

6. Dentali F, Donadini MP, Clark N, Crowther MA, Garcia D, Hylek E, et al. Brand name versus generic warfarin: a systematic review of the literature. Pharmacotherapy. 2011 Apr;31(4):386-93. PubMed PMID: 21449627. Epub 2011/04/01. eng.

7. Yacubian EM. Uso de medicamentos antiepilépticos genéricos, similares e de referência no tratamento das epilepsias. In: Guilhoto LM, Storpirtis S, editors. Atualização terapêutica: o impacto da diversificação de formulações de drogas antiepilépticas na prática clínica. São Paulo: Leitura Médica; 2010. p. 107-20.

8. Brasil. Lei nº 6360, de 23 de setembro de 1976. Dispõe sobre a Vigilância Sanitária a que ficam sujeitos os Medicamentos, as Drogas, os Insumos Farmacêuticos e Correlatos, Cosméticos, Saneantes e Outros Produtos, e dá outras Providências. Brasília: Diário Oficial da União. 24 setembro; 1976. p. 12647.

9. Storpirtis S. Princípios de biodisponibilidade, bioequivalência, equivalência farmacêutica e terapêutica de medicamentos. In: Guilhoto LM, Storpirtis S, editors. Atualização terapêutica: o impacto da diversificação de formulações de drogas antiepiléticas na prática clínica. São Paulo: Leitura Médica; 2010. p. 15-44.

10. Brasil. Ministério da Saúde. Agência Nacional de Vigilância Sanitária. Resolução RDC nº 16, de 02 de março de 2007. Regulamento técnico para medicamento similar. Brasília: Diário Oficial da União; 2007.

11. Approved products with therapeutic equivalence evaluations. In: US Department of Health and Human Services PHS, Food and Drug Administration, Center for Drug Evaluation and Research, Office of Pharmaceutical Sciences, Office of Generic Drugs, editor. 33nd ed. Washington, DC, 2013.

12. Canada. Minister of Health. Health Canada. Comparative Bioavailability Standards: Formulations Used for Systemic Effects. Ottawa, ON2012. p. 11.

13. Neutel JM, Smith DH. A randomized crossover study to compare the efficacy and tolerability of Barr warfarin sodium to the currently available Coumadin®. CVR&R. 1998:49-59.

14. Weibert RT, Yeager BF, Wittkowsky AK, Bussey HI, Wilson DB, Godwin JE, et al. A randomized, crossover comparison of warfarin products in the treatment of chronic atrial fibrillation. The Annals of pharmacotherapy. 2000 Sep;34(9):981-8. PubMed PMID: 10981241. Epub 2000/09/12. eng.

15. Pereira JA, Holbrook AM, Dolovich L, Goldsmith C, Thabane L, Douketis JD, et al. Are brand-name and generic warfarin interchangeable? Multiple n-of-1 randomized, crossover trials. The Annals of pharmacotherapy. 2005 Jul-Aug;39(7-8):1188-93. PubMed PMID: 15914517. Epub 2005/05/26. eng.

16. Lee HL, Kan CD, Yang YJ. Efficacy and tolerability of the switch from a branded to a generic warfarin sodium product: an observer-blinded, randomized, crossover study. Clinical therapeutics. 2005 Mar;27(3):309-19. PubMed PMID: 15878384. Epub 2005/05/10. eng.

17. Henderson JD, Esham RH. Generic substitution: issues for problematic drugs. Southern medical journal. 2001 Jan;94(1):16-21. PubMed PMID: 11213935. Epub 2001/02/24. eng.

18. Paveliu MS, Bengea S, Paveliu FS. Generic Substitution Issues: Brand-generic Substitution, Generic-generic Substitution, and Generic Substitution of Narrow Therapeutic Index (NTI)/Critical Dose Drugs. Maedica. 2011 Jan;6(1):52-8. PubMed PMID: 21977191. Pubmed Central PMCID: PMC3150029. Epub 2011/10/07. eng.

19. Richton-Hewett S, Foster E, Apstein CS. Medical and economic consequences of a blinded oral anticoagulant brand change at a municipal hospital. Archives of internal medicine. 1988 Apr;148(4):806-8. PubMed PMID: 3355300. Epub 1988/04/01. eng.

20. Swenson CN, Fundak G. Observational cohort study of switching warfarin sodium products in a managed care organization. American journal of health-system pharmacy : AJHP : official journal of the American Society of Health-System Pharmacists. 2000 Mar 1;57(5):452-5. PubMed PMID: 10711525. Epub 2000/03/11. eng.

21. Witt DM, Tillman DJ, Evans CM, Plotkin TV, Sadler MA. Evaluation of the clinical and economic impact of a brand name-to-generic warfarin sodium conversion program. Pharmacotherapy. 2003 Mar;23(3):360-8. PubMed PMID: 12627935. Epub 2003/03/12. eng.

22. Leite FQ. Intercambialidade entre medicamentos genéricos: estudo de caso fluoxetina. São Paulo: Universidade Federal de São Paulo. Escola Paulista de Medicina.; 2013.

23. Leiria TL, Pellanda L, Miglioranza MH, Sant'anna RT, Becker LS, Magalhaes E, et al. [Warfarin and phenprocoumon: experience of an outpatient anticoagulation clinic]. Arq Bras Cardiol. 2010 Jan;94(1):41-5. PubMed PMID: 20414525. Epub 2010/04/24. Varfarina e femprocumona: experiencia de um ambulatorio de anticoagulacao. por.

24. Zimerman LI, Fenelon G, Martinelli Filho M, Grupi C, Atié J, Lorga Filho A, et al. Diretrizes Brasileiras de Fibrilação Atrial. Arq Bras Cardiol. 2009;92(6 supl. 1):1-39.

25. Cheng A, Kumar K. Overview of atrial fibrillation. 2013 May 29, 2013.; 2013(30 June). Available from: http://www.uptodate.com/contents/overview-of-atrial-fibrillation?topicKey=CARD%2F1022&elapsedTimeMs=0&source=search_result&searchTerm=atrial+fibrillation&selectedTitle=1~150&view=print&displayedView=full.

26. Fuster V, Ryden LE, Cannom DS, Crijns HJ, Curtis AB, Ellenbogen KA, et al. ACC/AHA/ESC 2006 Guidelines for the Management of Patients with Atrial Fibrillation: a report of the American College of Cardiology/American Heart Association Task Force on Practice Guidelines and the European Society of Cardiology Committee for Practice Guidelines (Writing Committee to Revise the 2001 Guidelines for the Management of Patients With Atrial Fibrillation): developed in collaboration with the European Heart Rhythm Association and the Heart Rhythm Society. Circulation. 2006 Aug 15;114(7):e257-354. PubMed PMID: 16908781. Epub 2006/08/16. eng.

27. Singer DE, Chang Y, Fang MC, Borowsky LH, Pomernacki NK, Udaltsova N, et al. Should patient characteristics influence target anticoagulation intensity for stroke prevention in nonvalvular atrial fibrillation?: the

ATRIA study. Circulation Cardiovascular quality and outcomes. 2009 Jul;2(4):297-304. PubMed PMID: 20031854. Pubmed Central PMCID: PMC2801892. Epub 2009/12/25. eng.

28. Go AS, Mozaffarian D, Roger VL, Benjamin EJ, Berry JD, Borden WB, et al. Heart disease and stroke statistics--2013 update: a report from the American Heart Association. Circulation. 2013 Jan 1;127(1):e6-e245. PubMed PMID: 23239837. Epub 2012/12/15. eng.

29. Camm AJ, Lip GY, De Caterina R, Savelieva I, Atar D, Hohnloser SH, et al. 2012 focused update of the ESC Guidelines for the management of atrial fibrillation: an update of the 2010 ESC Guidelines for the management of atrial fibrillation. Developed with the special contribution of the European Heart Rhythm Association. European heart journal. 2012 Nov;33(21):2719-47. PubMed PMID: 22922413. Epub 2012/08/28. eng.

30. Lorga Filho AM, Azmus AD, Soeiro AM, Quadros AS, Avezum Junior A, Marques AC, et al. Diretrizes brasileiras de antiagregantes plaquetários e anticoagulantes em cardiologia. Arq Bras Cardiol. 2013 Setembro 2013;101(3 supl. 3):1-93.

31. Lip GY, Nieuwlaat R, Pisters R, Lane DA, Crijns HJ. Refining clinical risk stratification for predicting stroke and thromboembolism in atrial fibrillation using a novel risk factor-based approach: the euro heart survey on atrial fibrillation. Chest. 2010 Feb;137(2):263-72. PubMed PMID: 19762550. Epub 2009/09/19. eng.

32. Manning WJ, Singer DE, Lip GY. Antithrombotic therapy to prevent embolization in atrial fibrillation. 2013 30 June 2013. Available from: www.uptodate.com/contents/antithrombotic-therapy-to-prevent-embolization-in-atrial-fibrillation?source=see_link.

33. Hardman JG LL, Gilman AG. Anticoagulantes, trombolíticos e fármacos antiplaquetários. Goodman & Gilman, As bases farmacológicas da terapêutica. 10 ed. Rio de Janeiro: McGraw-Hill; 2005. p. 1141-58.

34. Marevan® varfarina sódica. Rio de Janeiro, RJ: Farmoquímica S/A; 2013.

35. Riley RS, Rowe D, Fisher LM. Clinical utilization of the international normalized ratio (INR). Journal of clinical laboratory analysis. 2000;14(3):101-14. PubMed PMID: 10797608. Epub 2000/05/08. eng.

36. Connolly SJ, Pogue J, Eikelboom J, Flaker G, Commerford P, Franzosi MG, et al. Benefit of oral anticoagulant over antiplatelet therapy in atrial fibrillation depends on the quality of international normalized ratio control achieved by centers and countries as measured by time in therapeutic range. Circulation. 2008 Nov 11;118(20):2029-37. PubMed PMID: 18955670. Epub 2008/10/29. eng.

37. Micromedex® 2.0.

38. Storpirtis S, Gonçalves JE, Chiann C, Gai MN. Biofarmacotécnica. Rio de Janeiro: Guanabara Koogan; 2011. 321 p.

39. Rosendaal FR, Cannegieter SC, van der Meer FJ, Briet E. A method to determine the optimal intensity of oral anticoagulant therapy. Thrombosis and haemostasis. 1993 Mar 1;69(3):236-9. PubMed PMID: 8470047. Epub 1993/03/01. eng.

40. Petersen P, Boysen G, Godtfredsen J, Andersen ED, Andersen B. Placebo-controlled, randomised trial of warfarin and aspirin for prevention of thromboembolic complications in chronic atrial fibrillation. The Copenhagen AFASAK study. Lancet. 1989 Jan 28;1(8631):175-9. PubMed PMID: 2563096. Epub 1989/01/28. eng.

41. Pocock SJ. The size of a clinical trial. Clinical trials: A practical approach: John Wiley & Sons; 1995. p. 266.

42. Warfarin Sodium. In: DrugPoints® System (electronic version). [Internet]. [cited 17 Julho 2013]. Available from: http://www.micromedexsolutions.com/.

ANNEX I

INFORMED CONSENT FORM

Research Title: Therapeutic equivalence between generic and branded warfarin sodium in adult patients with atrial fibrillation in Brazil - randomized crossover clinical trial

You are invited to participate as a volunteer in this research that aims to prove that the effect of the generic medicine warfarin is equivalent to that of the branded medicine (Marevan).

1. **Objectives:**

This research will verify if the substitution of the branded warfarin (Marevan) by the generic warfarins can cause important alteration in the results of laboratory tests of coagulation or change the quantity of undesirable clots (thromboembolic events) and bleeding events observed in the patients.

2. **Procedures and involvement of volunteers in the research:**

During this study, we will need you to return twice a month for 4 months to the outpatient Cardiology clinic at UNIFESP in order to: get the medication, do your laboratory exams (prothrombin time, PT, and the international normalized ratio, INR) and go through a follow-up consultation. You will also be asked to avoid starting any other treatment (with medicines prescribed by other doctors, self-medication or using teas and herbs) without first informing the study researcher, as this will hinder this research.

Usually we do not dispense warfarin at this outpatient clinic, but while participating in this research you will receive the medication here. All participants will receive both the branded medicine (Marevan) and the two generics (manufactured by the laboratories Teuto Brasileiro and União Química Farmacêutica Nacional), but neither you, nor the doctors or the pharmacist will know when you will receive each one, because the tablets are similar to each other and the packaging will be the same. This concealment is fundamental for the study to have reliable results.

3. **Risks and discomfort:**

Any patient using warfarin is at risk of minor bleeding (bleeding from the nose, gums, blood in the stool), but also severe bleeding (bleeding inside the skull, for example), which might require hospitalization or even cause death. To reduce this risk, it is necessary to do some laboratory tests which allows for the adjustment of the dose of warfarin. During these tests you may experience a temporary discomfort due to the need to draw blood from your arm vein.

Considering that, in this study, you will receive the usual treatment, the expected risk of bleeding or clotting is not very different from what other patients are submitted to, that is, a medium risk. Even so, we might find out that switching from the branded to the generic drug minimally increases this risk.

4. **Benefits:**

At the end of this study, you will know whether, for you, the effect of generic warfarin is the same as that of the brand medication. This will be important because, in case all the medications tested have the same effect, you might confidently decide to buy the generic drug, which is cheaper than the brand medication. The information provided by this study may also be applied to other patients similar to you, benefitting them as well.

5. **Confidentiality:**

The information we gather from you throughout this study will be pooled with that of other volunteers without disclosing any identifying information. The data and material collected will only be used for this research.

6. **Payment**

Payment will not be offered to study participants, but you will not have any expenses with the laboratory exams, medications, consultations and transportation related to this study.

______________________________ ______________________________

Participant's Signature Researcher's signature

***7.* Rights:**

You can decide not to participate in this research and if later you wish to give up and abandon the research at any time there will be no harm to your treatment at this clinic, with standard outpatient care being guaranteed. In case of personal injury directly caused by the procedures or treatments proposed in this study you will receive appropriate medical treatment, either in this outpatient clinic or at the São Paulo Hospital, as needed.

***8.* Contact:**

You have guaranteed access to the professionals responsible for the research to clarify any doubts. The main researcher is the pharmacist Carolina Gomes Freitas, who can be contacted by cell phone [Information omitted due to privacy concerns.] or found at the Federal University of São Paulo (UNIFESP), Rua Botucatu, 740, 3rd floor, PgMIT room (Graduate program in Internal Medicine and Therapeutics), Vila Clementino neighborhood in São Paulo / SP. If you have any concerns or doubts about the research ethics, please contact the Research Ethics Committee (CEP) - Rua Botucatu, 572 - 1º andar - cj 14, telephone 5571-1062, FAX: 5539-7162 - E -mail: cepunifesp@unifesp.br.

***9*. Informed consent**

I declare that, after being properly briefed by the researcher and having understood what was explained to me, I consent to participate in this Research Project. Two copies of this document must be signed, one remaining with you and the other with us. All the pages of this document are numbered and both the participant and the researcher must print their initials.

**Identification of the research subject**		
Name:		
ID:	Sex: male ( ) female ( )	
Date of birth:		Telephone:
Address:		

____

Participant's Signature

____

Witness signature (for illiterate volunteers)

I declare to comply with all the requirements described in this term.

____

Researcher's signature and ID   City/State: Date / /

ANNEX II

List of warfarin sodium interactions with drugs and food, according to severity (unknown, minor, moderate, major or contraindicated). DrugPoint Summary (42)

Contraindicated

Tamoxifen citrate

Major

abciximab	cephalothin sodium	enzalutamide	leflunomide	Papaya	teriflunomide
acenocumarol	cephapirin	eptifibatide	lepirudin	paroxetine	testosterone
alefacept	cephradine	erlotinib	levofloxacin	penicillin G	ticarcillin
amiodarone	Chamomile	erythromycin acistrate	lomitapide	penicillin V benzathine	ticlopidine
amoxicillin	ciprofloxacin	escitalopram	Lycium	pentosan polysulfate sodium	tinzaparin
ampicillin trihydrate	citalopram	etoposide	Marijuana	phenindione	tirofiban
apixaban	clarithromycin	etravirine	mechlorethamine	phenprocoumon	torsemide
aprepitant	clopidogrel	fenofibrate	methicillin	piperacillin	valproic acid
aspirin	cloxacillin benzathine	fenofibric acid	methotrexate	Pomegranate	venlafaxine
azythromycin	Cranberry juice	fibrinolytic agents	Methyl salicylate	posaconazole	vilazodone
bivalirudin	cyclophosphamide	Fish oil	metronidazole	prasugrel	vincristine
capecitabine	dabigatran	fluconazole	miconazole	procarbazine	vindesine
carbenicillin disodium	dabrafenib	fluoruracil	milnacipran	proguanil	voriconazole
carboplatin	dalteparin	fluoxetine	moxalactam	rivaroxaban	
cefadroxil	danaparoid	fluvoxamine	moxifloxacin	ropinirole	
cefdinir	dapsone	Garlic	nafcillin	roxithromycin	
cefepime	deferasirox	gatifloxacin	nalidixic acid	sertraline	
cefixime	desvenlafaxine	gemifloxacin	nandrolone	simvastatin	
cefotaxime	dicloxacillin	Ginkgo biloba	naproxen	sitaxsentan	
cefpodoxime	dipyridamole	imatinib	norfloxacin	St John's Wort	
ceftazidime	doxorubicin	infliximab	noscapine	sulfafurazol	
ceftibuten	dronedarone	Influenza virus vaccine	ofloxacin	sulfamethoxazole	
ceftizoxime	Drotrecogin alfa	itraconazole	oseltamivir	sulfisoxazole	
celecoxib	enoxacin	ketoconazole	oxacillin	Tan shen	
cephalexin	enoxaparin	ketoprofen	oxandrolone	telithromycin	

Moderate

Avocado	benzbromarone	ceftriaxone	desipramine	exenatide	Green tea
acarbose	betamethasona	Celery	Devil's claw	ezetimibe	griseofulvin
acemetacin	bicalutamide	Chaparral	dexamethasone	felbamate	Guggul
acetaminophen	Bilberry	chitosan	dexlansoprazole	fenbufen	halothane
Agrimony	bismuth subsalicylate	chloral hydrate	dexmethylphenidate	fenoprofen	heparin calcium
alclofenac	Black cohosh extract	chloramphenicol	dextropropoxifeno	Fenugreek	heptabarbital
Alfalfa	Black currant	chlordiazepoxide	diazoxide	Feverfew	hexobarbital sodium
allopurinol	Black haw	chlorotrianisene	diclofenac	floctafenine	High protein food
Aloe	Black tea	chlorpromazine hydrochloride	diethylstilbestrol	flosequinan	Horse chestnut
aminoglutethimide	Bladderwrack	cholestyramine	diflunisal	fludrocortisone acetate	Horseradish
amitriptyline	boceprevir	chondroitin	dipyrone	flufenamic acid	hydrocortisone
amobarbital sodium	Bogbean	cimetidine	disopyramide	fluoxymesterone	ibritumomab
amoxapine	Boldo	Cinchona	disulfiram	flurbiprofen	ibuprofen
amprenavir	Borage	cisapride monohydrate	Dong quai	flutamide	ifosfamide
Angelica	bosentan	cisplatin	dosulepina	fluvastatin	Imipramine hydrochloride
Anise	bromelain	clofibrate	dothiepin	fosamprenavir	indomethacin
Antithyroid agents	bromfenac	clomipramine	doxepin	fosaprepitant	indoprofen
apazone	Buchu	Clove	doxycycline calcium	Fucus vesiculosus	ipriflavone
aprobarbital	bufexamac	Clove oil	droxicam	gefitinib	isoniazid
argatroban	butabarbital	coenzyme Q10	duloxetine	gemcitabine	isoxicam
armodafinil	butalbital	colesevelam	Enteral nutrition	gemfibrozil	ivacaftor
Arnica	capsaicin	Contraceptives, combination	esomeprazole	Ginger	ivermectin
Asafetida	carbamazepine	cortisone	eterobarb	Ginseng, siberian	Kava
Astragalus	carprofen	Curcumin	ethacrynic acid	glimepiride	ketorolac tromethamine
atazanavir	Cassia	cyclosporine	Ethanol	glipizide	lactulose
atenolol	Cat's claw	danazol	ethchlorvynol	glucagon	lansoprazole
atovaquone	cefamandole	Dandelion	ethotoin	glucosamine	levamisole
azathioprine	cefazolin sodium	darunavir	etodolac	glutethimide	levotyroxine
Bee pollen	cefoperazone	delavirdine	etretinate	glyburide	Licorice
benoxaprofen	cefotetan	demeclocycline	Evening primrose oil	Goldenseal	lopinavir

lornoxicam	Noni juice	propiltiouracil	secobarbital	Tonka
lovastatin	Nortriptyline hydrochloride	propoxyphene	Senega	toremifene citrate
Meadowsweet	omega-3-acid ethyl esters	propranolol	Skullcalp	tramadol
meclofenamate	omeprazole	propyphenazone	sorafenib	trastuzumab
mefenamic acid	Onion oil	proquazona	Soybean	treprostinil
melatonin	orlistat	protriptyline	spironolactone	triamcinilone acetonide
meloxicam	oxaprozin	Pumpkin seed	stanozolol	trimipramine
menthol	oxiphenbutazone	Quassia	sucralfate	valdecoxib
mephobarbital	oxymetholone	quetiapine	sulfasalazine	vancomycin
mercaptopurine	oxytetracycline	quinestrol	sulfinpyrazone	vemurafenib
mesalamine	pantoprazole	quinidine	sulindac	vitamin A
Mesna	paramethasone	quinine	sulofenur	vitamin E
methandrostenolone	Parsley	rabeprazole sodium	suprofen	vitamin K (phytonadione)
methylphenidate	Passion flower	raloxifene	Sweet woodruff	Vitamin K containing food
methylprednisolone acetate	Pau d'arco	ranitidine	Tamarindo	vorinostat
methyltestosterone	pentoxifylline	Red clover	teduglutide	Wild lettuce
mifepristone	phenobarbital	rifabutin	telaprevir	Willow
minocycline	phenylbutazone	rifampin	tenidap sodium	Wintergreen
Mistletoe	phenytoin	rifapentine	tenoxicam	Yarrow
mitotane	piracetam	rifaximin	terbinafine	zafirlukast
moricizine	pirazolac	rilonacept	tetracycline	zileuton
Motherwort	piroxicam	ritonavir	Thyroid hormones	zomepirac sodium
nabumetone	pirprofen	rofecoxib	tiaprofenic acid	zotepine
nelfinavir	policosanol	romidepsin	tibolone	
neomycin	Poplar	rosuvastatin	ticlopidine	
Nettle extract	prednisolone	Salicylates	tigecycline	
nevirapine	prednisone	Salsaparilla	tinidazole	
niacin	Prickly ash	saquinavir	tocilizumab	
nilutamide	primidone	Saw palmetto extract	tolmetin	
nimesulide	propafenone hydrochloride	secbutabarbital	tolterodine	
