# Supplementary material for: Examining therapeutic equivalence between branded and generic warfarin in Brazil: The WARFA crossover randomized controlled trial
Source: PLoS One. 2021 Apr 1;16(4):e0248567. doi: 10.1371/journal.pone.0248567 (PMC8016229; doi:10.1371/journal.pone.0248567)
Supplement: S2 Protocol — (PDF) [file pone.0248567.s024.pdf]

Equivalência terapêutica entre a varfarina sódica de referência e as genéricas em pacientes adultos com fibrilação atrial no Brasil - ensaio clínico *crossover* randomizado

Pesquisador(a) Principal: Carolina Gomes Freitas

Orientador(a): Prof. Dr. Álvaro Nagib Atallah

Co-orientador(a): Dr. Dirceu Raposo de Mello

LISTA DE ABREVIATURAS

ANVISA	Agência Nacional de Vigilância Sanitária
ASC	area sob a curva
AVC	acidente vascular cerebral
Cmáx	concentração máxima
FA	fibrilação atrial
FDA	*Food and Drug Administration*
HU-USP	Hospital Universitário da Universidade de São Paulo
INR	*international normalized ratio*
ISI	*international sensitivity índex*
SUS	Sistema Único de Saúde
TCLE	termo de consentimento livre e esclarecido
TP	tempo de protrombina
TTR	*time in therapeutic range*
UNIFESP	Universidade Federal de São Paulo
USP	Universidade de São Paulo
UTN	Universal Trial Number

1. Fundamentos e razões

1.1 Medicamentos genéricos

No Brasil o medicamento genérico está incluído dentre as diretrizes e prioridades da Política Nacional de Medicamentos (1). Há 13 anos no mercado brasileiro (2), o genérico encontra-se efetivamente incorporado ao Sistema Único de Saúde (SUS), à Saúde Complementar e ao cotidiano da população, sendo responsável no primeiro semestre de 2013, segundo dados do *IMS Health*, pelo valor de R$ 6,3 bilhões em vendas (3) e por 27,15% em unidades vendidas (4) no mercado farmacêutico brasileiro. O genérico assume assim papel importante no país, embora ainda seja objeto de controvérsia inclusive entre os profissionais da saúde e pesquisadores, especialmente em se tratando de medicamentos antiepiléticos, imunossupressores e de baixo índice terapêutico (5-7). Questiona-se se para essas classes de medicamentos os critérios para registro de genéricos são suficientes para garantir que estes e os medicamentos de referência sejam realmente equivalentes terapêuticos e consequentemente intercambiáveis.

Para que um medicamento seja comercializado no Brasil há exigência de registro concedido pela Agência Nacional de Vigilância Sanitária (ANVISA) mediante comprovação de eficácia, segurança e qualidade do produto (8). Para um medicamento inovador, ou seja, inédito, a comprovação da eficácia e segurança é realizada por meio de ensaio clínico. Para o medicamento genérico, que é similar a um medicamento de referência (ou inovador) já registrado, a comprovação da eficácia e segurança exige apenas o estudo de bioequivalência (9). Considera-se que a segurança e eficácia do fármaco genérico foram comprovadas anteriormente pelo ensaio clínico conduzido para registro do medicamento de referência, bastando apenas o atestado de que as biodisponibilidades (quantidade de medicamento absorvida e taxa de absorção) dos medicamentos não apresentam diferenças estatisticamente significantes quando administrados nas mesmas condições experimentais à mesma dose molar do princípio ativo (10). Em parâmetros farmacocinéticos, a quantidade de medicamento absorvida é representada pela área sob a curva (ASC) e a taxa de absorção, pela concentração máxima (Cmáx).

O critério adotado no Brasil e internacionalmente (9) para classificação do medicamento como bioequivalente ao de referência, condição para registro do medicamento como genérico, baseia-se na premissa de que, para a maior parte dos medicamentos, uma variação de 20% para mais ou para menos na concentração do fármaco no sangue não seria clinicamente significante (5, 11). Em termos estatísticos, isto exige que os valores extremos do intervalo de confiança de 90% para a razão das médias geométricas da $ASC_{teste}/ASC_{referência}$ e das $Cmáx_{teste}/Cmáx_{referência}$ devem ser maiores que 0,8 e menores que 1,25, sendo teste a denominação do medicamento genérico; ou seja, o intervalo de confiança da relação entre os parâmetros farmacocinéticos do medicamento genérico em relação ao de referência deve estar contido dentro do limite de 80% a 125% (7).

Algumas agências sanitárias, contudo, estabelecem critérios adicionais, mais rígidos para determinados medicamentos: a Health Canada, por exemplo, estabelece o limite de 90% a 112% para o intervalo de confiança referente à ASC, para determinados fármacos críticos, assim classificados por serem medicamentos nos quais pequenas variações na dose ou na concentração podem desencadear graves eventos adversos que poderiam ser fatais, deixar sequelas ou requerer hospitalização. Dentre esses fármacos encontra-se a varfarina (12). Desse modo, o conceito amplamente assumido de que a varfarina possui baixo índice terapêutico (6, 12, 13) levou ao questionamento por parte dos profissionais de saúde sobre se os parâmetros normalmente adotados para bioequivalência efetivamente garantem equivalência terapêutica entre o medicamento de referência e os genéricos desse fármaco (6, 13-18).

Dentali (6), em revisão sistemática, constatou que estudos observacionais suscitaram os resultados mais preocupantes, como a grande diferença na porcentagem de exames de protrombina (PT) dentro do intervalo de referência entre 15 pacientes que realizaram a troca para o medicamento genérico em relação a 40 controles que

mantiveram o medicamento de referência (39% e 69%)(19). Entretanto, os resultados foram conflitantes (6), com outros estudos não demonstrando alterações na média do exame de coagulação razão normalizada internacional (INR, da sigla em inglês) em pacientes que voluntariamente trocaram para o medicamento genérico (105 sujeitos) quando em comparação aos que mantiveram o de referência (outros 105 sujeitos, INR 2,7 vs. 2,8)(20); ou ainda identificando diminuição estatisticamente significante, mas não clinicamente significante, da média do tempo dentro do intervalo de referência (TTR, da sigla em inglês para *time in therapeutic range*) em coorte de 2299 pacientes que substituíram a varfarina de referência pela genérica (65,9% vs. 63.3%)(21).

Os ensaios clínicos, por outro lado, obtiveram resultados mais homogêneos (6). Desenhos de estudo randomizados e cruzados (13-16) comparando a substituição de varfarina de referência pela genérica não demonstraram diferenças estatisticamente significantes em diversos desfechos avaliados nas diferentes formulações do medicamento, porém ao menos duas grandes limitações no âmbito da validade interna desses trabalhos podem ser apontadas: os desenhos de estudo sujeitos a efeito *carry-over* (13-16) e a exclusão de substancial número de pacientes da análise final por terem necessitado de ajuste de dose do medicamento (13). Há de se levar em conta ainda aspectos da validade externa dos estudos, uma vez que nenhum deles foi realizado no Brasil.

Além da substituição entre o medicamento de referência pelo genérico, outra questão preocupante é a da substituição entre as diversas formulações de genéricos entre si, situação comum na prática clínica. Já foi demonstrado para as formulações disponíveis no mercado brasileiro do medicamento antidepressivo fluoxetina que, embora os genéricos fossem bioequivalentes ao medicamento de referência, sendo, portanto, intercambiáveis do ponto de vista legal, nem todos seriam bioequivalentes entre si (22). Teoricamente isso poderia acarretar consequências clínicas, como diminuição da eficácia ou manifestação de efeitos adversos caso o paciente realize a troca entre formulações não bioequivalentes entre si.

Deste modo, este trabalho se propõe a realizar um ensaio clínico *crossover* randomizado para avaliar se a substituição da varfarina sódica da marca de referência (Marevan®, União Química/Farmoquímica) por qualquer uma das formulações genéricas disponíveis no mercado brasileiro (fabricadas pelos laboratórios União Química Farmacêutica Nacional S/A e Laboratório Teuto Brasileiro S/A) e, entre as próprias formulações genéricas entre si, é efetiva e segura para o paciente com fibrilação atrial. Este tipo de paciente foi escolhido porque fibrilação atrial figura entre as indicações mais prevalentes (23) para o uso deste anticoagulante oral nos ambulatórios especializados.

1.2 Fibrilação atrial

A fibrilação atrial (FA) é uma taquiarritmia que promove desorganização da atividade elétrica atrial, diminuindo assim a capacidade de contração e consequentemente dificultando a sístole atrial (24). Caracteriza-se ao eletrocardiograma pela ausência de ondas P claramente definidas e pela ausência de um padrão repetitivo do intervalo RR (25). FA é a arritmia mais comum na prática clínica, sendo responsável por aproximadamente um terço das hospitalizações por distúrbios do ritmo cardíaco. Estima-se prevalência de 0,4% a 1% na população geral, aumentando com a idade, sendo 8% em pacientes com idade maior de 80 anos (26).

Os pacientes normalmente acometidos são idosos do sexo masculino, os quais, em decorrência da doença, podem sofrer com diminuição do débito cardíaco e maiores riscos de tromboembolismo de origem atrial e morte (25). Dentre os eventos tromboembólicos, aproximadamente 90% são acidentes vasculares cerebrais (AVC) (27), principais responsáveis pela invalidez de pacientes nos Estados Unidos (28). O risco de AVC nos pacientes com FA é em média 5% ao ano, cerca de duas a sete vezes maior do que em pessoas sem FA; e o risco de morte é aproximadamente o dobro (26). O efeito incapacitante do AVC pode ser observado em uma coorte de pacientes do estudo *Framingham* com idade igual ou superior a 65 anos que sobreviveram a esse evento; após seis meses do episódio as seguintes sequelas puderam ser observadas: 50% dos pacientes possuíam hemiparesia; 46% possuíam déficits cognitivos; 30% eram incapazes de andar sem algum tipo de auxílio e 19% estavam afásicos (28).

A FA pode ser classificada em valvar, quando é relacionada à doença valvular reumática ou a próteses valvares mecânicas, e não valvar (29). Todos os pacientes com FA possuem indicação para terapia antitrombótica, contudo a classe de medicamentos indicada (antiplaquetários ou anticoagulantes) depende da relação entre o risco de sangramentos e o benefício em termos de prevenção de eventos trombóticos, especialmente AVC (30). Atualmente, a estratificação de risco dos pacientes para tromboembolismo na FA é realizada pelo escore validado CHA_2DS_2VASc, acrônimo em inglês para os fatores de risco envolvidos e sua respectiva pontuação individual: insuficiência cardíaca congestiva ou fração de ejeção do ventrículo esquerdo ≤ 40, hipertensão arterial sistêmica, idade maior ou igual a 75 anos, diabetes mellitus, AVC ou acidente isquêmico transitório ou evento tromboembólico prévios, doença vascular (incluindo infarto do miocárdio, placas ateroscleróticas na aorta e doença arterial periférica), idade entre 65 e 74 anos e gênero feminino. Os fatores de risco definitivos (idade acima de 75 anos e eventos trombóticos prévios) recebem pontuação dois, enquanto os outros fatores recebem pontuação um; o resultado final varia de zero a dez e pacientes com pontuação final zero, um, ou maior ou igual a dois são considerados, respectivamente, de baixo, moderado e alto risco para AVC (31). Na ausência de contraindicações, pacientes com risco moderado e alto (escore final igual ou maior que um) devem receber anticoagulação oral (30).

Apesar da aprovação dos novos anticoagulantes orais, rivaroxabana e dabigatrana, nos últimos dois anos pela Agência Nacional de Vigilância Sanitária (ANVISA) para uso nessa condição, a varfarina, anticoagulante de primeira linha, ainda deverá ter uso muito difundido por vários anos considerando o custo muito elevado desses novos medicamentos e o pequeno benefício em segurança e eficácia apresentado relativamente terapia tradicional (32).

1.3 Varfarina

A varfarina é um fármaco sintético do grupo das cumarinas, antagonistas da vitamina K com efeito anticoagulante. Seu mecanismo de ação envolve os fatores de coagulação II, VII, IX, X e as proteínas anticoagulantes C e S, cujas ativações dependem da vitamina K. Essa ativação ocorre por meio de reação de carboxilação e exige que a vitamina K esteja em sua forma reduzida, resultando em vitamina K oxidada (epóxido). A vitamina K reduzida necessária pode ser obtida de fonte externa (após ingestão da vitamina K e redução por outra enzima redutora, a DT-diaforase) ou por regeneração da vitamina K oxidada por meio da enzima vitamina K epóxido-redutase, a qual é inibida pelas cumarinas. A administração da varfarina nas doses terapêuticas diminui não apenas a atividade biológica dos fatores de coagulação para 10-40% do normal como também a quantidade total de cada um dos fatores dependentes de vitamina K sintetizados pelo fígado em 30-50%. Contudo, como os fatores de coagulação ativados na circulação não são afetados pela varfarina, o efeito antitrombótico total só é atingido após a depuração destes depois de alguns dias. (33)

A molécula possui enantiômeros, mas normalmente é administrado como mistura racêmica, não tendo sido identificada vantagem da administração de um único enantiômero. O fármaco possui alta biodisponibilidade quando administrado por via oral, a via de escolha para administração; é metabolizado pelo fígado a metabólitos inativos e excretado na urina e fezes. O tempo de meia-vida de eliminação varia de 25 a 60 horas e a duração da ação, de 2 a 5 dias. (33)

É um fármaco teratogênico, classificado como categoria X pelo FDA (*Food and Drug Administration*) e, de acordo com o fabricante, não é indicado para lactantes (34). Durante o primeiro trimestre da gravidez pode induzir má-formação fetal (34), síndrome caracterizada por hipoplasia nasal e calcificações epifisárias pontilhadas; durante o segundo e terceiro trimestres também já foram relatadas anormalidades do sistema nervoso central (33) e, em estágios mais avançados da gravidez, está associada à hemorragia fetal e aumento da taxa de aborto (34) mesmo quando os valores de tempo de protrombina (TP) da gestante estão dentro da faixa terapêutica (33).

A presença de alimento no trato gastrintestinal pode diminuir a taxa de absorção da varfarina e uma extensa lista de fármacos pode alterar a ação dos inibidores de vitamina K. São substâncias ou situações potencialmente perigosas aquelas que alterem: a biodisponibilidade ou o metabolismo do anticoagulante oral ou da vitamina K; a síntese, a função ou a depuração de qualquer fator ou célula envolvidos na hemostasia ou na fibrinólise; ou a integridade de qualquer superfície epitelial. São exemplos dessas condições: a redução do efeito anticoagulante devido ao aumento da depuração da varfarina secundariamente à indução de enzimas hepáticas por ingestão crônica de álcool; o aumento do risco de hemorragia por inibição da função plaquetária decorrente do uso de ácido acetilsalicílico; o aumento do risco de hemorragia por dieta pobre em vitamina K especialmente quando associada à depleção da flora intestinal, que produz vitamina K, pelo uso de antibióticos; e o aumento do risco de hemorragia por gastrite ou ulceração induzida por anti-inflamatórios não esteroidais (33).

A dose recomendada para início da terapia é de 2,5 a 5,0 mg e, para manutenção, de 2,5 a 10 mg por dia com a dose ajustada de acordo com os resultados obtidos nos exames de TP e INR, a medida normalizada do TP (34). O TP mensura o tempo necessário para a coagulação do sangue a partir da adição do reagente tromboplastina e do cálcio, que ativam a coagulação pela via extrínseca, sendo sensível aos fatores II, V, VII e X de coagulação (35). Ele é expresso em segundos ou como a relação entre o tempo de coagulação do paciente e o de uma média de plasma normais (35). A origem da tromboplastina (tecido cerebral humano, tecido cerebral de coelho, de porco, recombinante etc) determina sua sensibilidade, o que, em adição a outras variáveis inter e intralaboratoriais, pode acarretar alterações do TP da ordem de dez segundos ou mais. Essa variação motivou a adoção do INR, que considera a sensibilidade do lote de tromboplastina em relação a um padrão de referência internacional (ISI, *international sensitivity index*), como medida padronizada do TP. (35) O cálculo do INR é realizado da seguinte forma:

$$INR = \left(\frac{TP\ paciente}{média\ geométrica\ TP\ normal}\right)^{ISI} = (relação\ TP)^{ISI}$$

Assim, para pacientes com fibrilação atrial, o intervalo terapêutico alvo do INR é estabelecido entre 2,0 e 3,0 visando otimizar a relação entre o risco de hemorragia intracraniana e o benefício da prevenção de eventos tromboembólicos (27). Evidências de estudos observacionais corroboram esse intervalo. Em particular, um estudo de caso-controle aninhado em uma coorte de 13559 pacientes adultos com fibrilação atrial não-valvular no norte da Califórnia, nos Estados Unidos, identificou 397 casos de eventos tromboembólicos e 164 casos de hemorragia intracraniana e demonstrou que, em relação ao INR dentro do intervalo de 2,0 a 2,5, a redução do INR para abaixo de 1,8 aumenta acentuadamente o risco de eventos tromboembólicos; e que o aumento do INR acima de 3,5 aumenta de modo marcante o risco de hemorragia intracraniana. (27)

Nem todos os pacientes, entretanto, conseguem atingir e manter o intervalo terapêutico do INR. Análise *posthoc* do tempo dentro do intervalo terapêutico (TTR, da sigla em inglês) médio atingido pelos diferentes centros e países de um ensaio clínico multicêntrico demonstrou grande heterogeneidade nos valores (TTR entre 46,3% na África do Sul e 77,8% na Suécia) o que impactou na magnitude do benefício obtido pela terapia com anticoagulantes orais (36). Especialistas estimam que um TTR de ao menos 60% seja necessário para obtenção de maior benefício com a varfarina, sendo cada vez maior o benefício esperado do fármaco quanto maior o TTR (32).

2. Objetivos

2.1 Objetivo geral

Determinar, por meio da média de INR dos pacientes, se há equivalência terapêutica entre a varfarina sódica de referência e as formulações genéricas em pacientes com fibrilação atrial no Brasil.

2.2 Objetivos específicos

Avaliar, quando em uso das diferentes formulações de varfarina:

- a média de INR do paciente;
- a média do TP;
- a incidência de eventos tromboembólicos;
- a incidência de sangramentos;
- o TTR;
- a adesão ao tratamento.

3. Hipótese

A média do INR dos pacientes quando em uso das formulações genéricas de varfarina não difere mais que 0,49 (bicaudal, com nível de significância de 5%) da média de INR observada com o medicamento de referência, demonstrando equivalência terapêutica entre eles.

4. Métodos

4.1 Tipo de estudo

Ensaio clínico randomizado *crossover* contendo seis grupos com sequências de tratamento diferentes e dividido em quatro fases com durações diferentes (Figura 1). O estudo está registrado em www.clinicaltrials.gov sob a identificação NCT02017197 e possui ainda o UTN U1111-1155-4833.

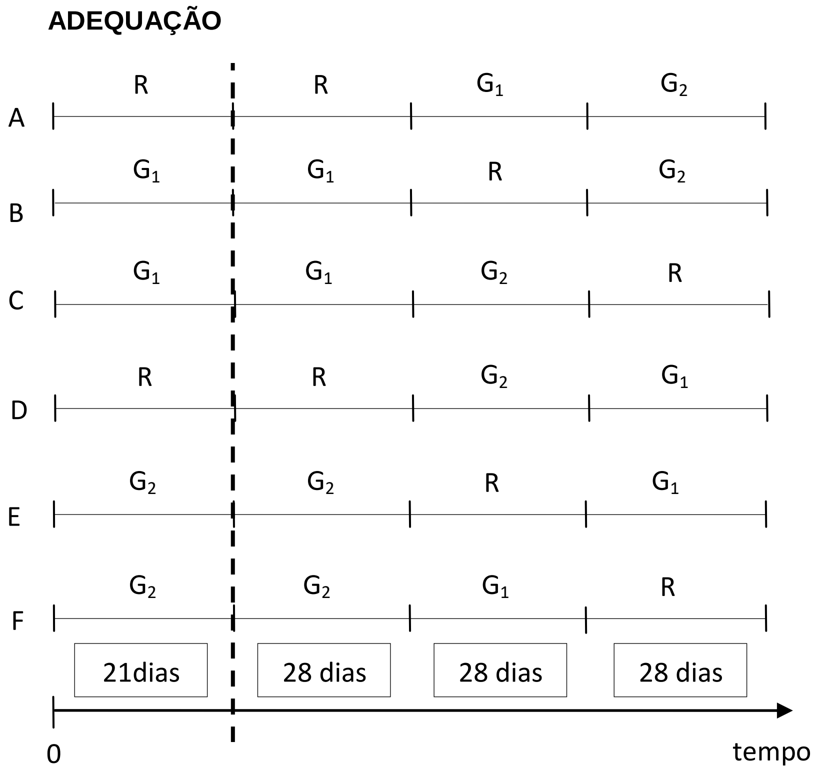

Figura 1: Representação das sequências de tratamento para os pacientes randomicamente alocados nos grupos A, B, C, D, E ou F em relação ao tempo. R: medicamento de referência. G1 e G2: medicamentos genéricos de marcas diferentes.

4.2 Local

Ambulatórios da Disciplina de Cardiologia, do Departamento de Medicina, da Escola Paulista de Medicina, na Universidade Federal de São Paulo.

4.3 Participantes

4.3.1 Critérios de inclusão

Serão incluídos no estudo os pacientes que, de acordo com entrevista estruturada ou por consulta ao prontuário, atendam simultaneamente todos os critérios:

- diagnóstico de fibrilação atrial (por eletrocardiograma) (26) não valvar (por ecocardiograma);
- com indicação para uso de varfarina por resultado maior ou igual a um no escore CHA2DS2VASc;
- em uso de varfarina;
- com idade acima de 18 anos;
- que concordem em participar do estudo informando consentimento por meio de assinatura do Termo de Consentimento Livre e Esclarecido (TCLE) (Anexo I).

4.3.2 Critérios de exclusão

Não serão aceitos pacientes que se encaixem em quaisquer dos critérios:

- com contraindicação para o uso de anticoagulantes (pacientes em uso de outros anticoagulantes, hemorragia ativa, hipersensibilidade à varfarina, úlceras);
- mulheres em idade fértil, grávidas ou lactantes;
- pacientes com trombocitopenia;
- pacientes com insuficiência hepática ou renal;
- pacientes com histórico de episódios hemorrágicos por deficiência congênita de fatores de coagulação;
- pacientes voluntários em outros ensaios clínicos;
- pacientes que na fase de adequação do estudo não atinjam TTR de pelo menos 70% (serão considerados não-aderentes à terapia);
- pacientes iniciando tratamento com medicamentos que interajam de modo moderado a importante ou sejam contraindicados quando utilizados concomitantemente com a varfarina, de acordo com a base de dados *online* Micromedex® 2.0 (37) (Anexo II).

4.4 Procedimentos

A primeira fase do estudo (Figura 1), de adequação, terá a duração de 21 dias. Nela os pacientes, após randomização e consequente alocação a uma das sequências de tratamento (A, B, C, D, E e F), iniciarão o tratamento com a formulação de varfarina da marca de referência (denominada R) ou com uma das duas formulações de varfarina sódica genérica disponíveis no mercado brasileiro em agosto de 2013 (G_1 e G_2). Neste período, o paciente, que já estava em uso de varfarina, iniciará o tratamento que será mantido na segunda fase do ensaio clínico, tendo por objetivo evitar o efeito *carry-over* sem a interrupção do tratamento, que seria eticamente inaceitável. A duração prevista de três semanas para esta fase é equivalente a cerca de sete meias-vidas, tempo de *washout (38)*, para eliminação do fármaco utilizado anteriormente; assim será possível garantir que a formulação de varfarina utilizada anteriormente não interfira nos resultados do ensaio.

A segunda, terceira e quarta fases terão duração de 28 dias cada, a fim de permitir mais mensurações semanais de avaliação do efeito da terapia com cada uma das formulações. Na segunda fase os pacientes manterão o tratamento iniciado na fase um, e na terceira e quarta fases ocorrerá a troca das terapias de acordo com as sequências predeterminadas para cada grupo.

4.4.1 Randomização

A sequência de randomização será gerada por um investigador independente do estudo (TFCP) utilizando uma tabela de números aleatórios produzida com o *software* Microsoft Excel. A medicação será separada e dispensada pela investigadora principal (CGF). O investigador independente (TFCP) será o responsável pela manutenção do sigilo da sequência até a fase de análise dos dados, atribuindo à medicação separada as iniciais do paciente e um código alfanumérico que correlacionará com a formulação da varfarina utilizada.

4.4.2 Intervenção

Os pacientes receberão, de acordo com a fase do estudo, o medicamento varfarina sódica da marca de referência (R, Marevan®, fabricado por União Química/Farmoquímica, Brasil) ou um dos medicamentos genéricos (G_1, União Química Farmacêutica Nacional S/A ou G_2, Laboratório Teuto Brasileiro S/A, Brasil) disponíveis no mercado brasileiro em agosto de 2013.

A prescrição do medicamento será realizada pela denominação genérica, permitindo assim que o paciente receba tanto o medicamento de referência como as diversas formulações de genérico. Os comprimidos disponíveis no mercado possuem aparência semelhante entre si e a dispensação será realizada pela investigadora principal no próprio ambulatório em embalagens opacas e iguais, visando assim impedir que pacientes, médicos e a investigadora principal tenham conhecimento da formulação de varfarina que o paciente utilizará. Será dispensada medicação suficiente para o período entre os exames laboratoriais. Posteriormente será realizada a contagem dos comprimidos retornados com dois objetivos: avaliar e estimular a adesão ao tratamento. O ajuste de dose será realizado mensalmente, ou mais frequentemente a critério médico, caso o INR resulte fora do intervalo de referência, com base no resultado do INR, cujo alvo é a manutenção entre 2,0 e 3,0.

4.5 Variáveis e desfechos

Os dados dos desfechos serão coletados pela investigadora principal (CGF) sendo os exames laboratoriais (INR e TP) e os desfechos clínicos (eventos tromboembólicos e sangramentos) avaliados duas vezes por mês; a adesão ao tratamento será avaliada mensalmente.

4.5.1 Desfecho primário

- Média do INR

  Será comparada a média do INR de cada paciente quando em uso das diversas formulações de varfarina.

4.5.2 Desfechos secundários

- Média do TP

  A média do TP será avaliada do mesmo modo que o INR.
- Tempo dentro do intervalo terapêutico (TTR).

  Esta relação é calculada para cada paciente pela porcentagem de pessoa-tempo dentro do intervalo terapêutico de 2,0 e 3,0 dividido pela contribuição total em pessoa-tempo desse mesmo indivíduo (39).
- Incidência de eventos tromboembólicos

  Inclui acidente vascular cerebral (AVC) isquêmico (diferenciado do AVC hemorrágico por tomografia) e tromboembolismo em vísceras ou extremidades (diagnosticado por sintomas agudos e testes diagnósticos relevantes) (40).
- Incidência de sangramentos

  Os sangramentos serão classificados como episódios hemorrágicos ou sangramentos menores:
  - Hemorragia grave: hemorragia intracraniana, hemorragia fatal, perda de sangue que resulte em diminuição maior ou igual a 2,0 g/L da hemoglobina sérica, hemorragia que exija transfusão, sangramento em áreas sensíveis tais como retina ou pericárdio.
  - Sangramentos menores: todos os outros sangramentos (33).

- Aderência ao tratamento

  Será mensurada pela quantidade de comprimidos retornados pelo paciente.

4.6 Método Estatístico

4.6.1 Tamanho da amostra

Serão necessários no mínimo 48 pacientes (8 em cada grupo) a fim de verificar uma diferença clinicamente significante de 0,49 no INR, considerando-se que é esperada uma média e desvio padrão de INR com a varfarina de referência de 2,45 e 0,29, respectivamente (13). A amostra foi calculada com base no desfecho de média de INR, utilizando o método estatístico para desfechos quantitativos (41) assumindo-se α=5% e β=10%, que resultam em um poder de 90%. Para compensação de possíveis desistências ou exclusões de pacientes, planeja-se a adesão de 60 indivíduos (10 em cada grupo).

4.6.2Análise estatística

Todas as análises estatísticas terão nível de significância de 5%. Para o desfecho de média do INR e TP será utilizado teste–t de Student pareado. Para estes desfechos será realizada análise por protocolo, ou seja, serão excluídos da análise final todos os dados de pacientes que durante o estudo iniciem tratamento com algum dos fármacos ou substâncias que interagem de modo moderado a importante com a varfarina (relação no anexo II).

Os desfechos dicotômicos (TTR, eventos tromboembólicos, sangramentos) serão analisados por teste de McNemar. Não são previstas análises exploratórias para subgrupos de pacientes. As variáveis dicotômicas serão analisadas por intenção de tratar, considerando-se os dados faltantes como desfecho desfavoráveis e nesse caso será realizada análise de sensibilidade para avaliação da robustez do resultado.

5. Referências bibliográficas

1. Brasil. Ministério da Saúde. Política Nacional de Medicamentos. Portaria Nº 3.916, de 30 de outubro de 1998. Brasília: Diário Oficial da União; 1998.

2. Brasil. Ministério da Saúde. Agência Nacional de Vigilância Sanitária. Medicamento Genérico. Brasília.

3. PróGenéricos. Associação Brasileira das Indústrias de Medicamentos Genéricos. Vendas de genérico chegam a 6,3 bi 2013 [updated 26 Julho 2013; cited 2013 19 Agosto]. Available from: http://www.progenericos.org.br/index.php/noticias/328-vendas-de-generico-chegam-a-r-63-bi.

4. PróGenéricos. Associação Brasileira das Indústrias de Medicamentos Genéricos. Mercado [cited 2013 19 Agosto]. Available from: http://www.progenericos.org.br/index.php/mercado.

5. Davit BM, Nwakama PE, Buehler GJ, Conner DP, Haidar SH, Patel DT, et al. Comparing generic and innovator drugs: a review of 12 years of bioequivalence data from the United States Food and Drug Administration. The Annals of pharmacotherapy. 2009 Oct;43(10):1583-97. PubMed PMID: 19776300. Epub 2009/09/25. eng.

6. Dentali F, Donadini MP, Clark N, Crowther MA, Garcia D, Hylek E, et al. Brand name versus generic warfarin: a systematic review of the literature. Pharmacotherapy. 2011 Apr;31(4):386-93. PubMed PMID: 21449627. Epub 2011/04/01. eng.

7. Yacubian EM. Uso de medicamentos antiepilépticos genéricos, similares e de referência no tratamento das epilepsias. In: Guilhoto LM, Storpirtis S, editors. Atualização terapêutica: o impacto da diversificação de formulações de drogas antiepilépticas na prática clínica. São Paulo: Leitura Médica; 2010. p. 107-20.

8. Brasil. Lei nº 6360, de 23 de setembro de 1976. Dispõe sobre a Vigilância Sanitária a que ficam sujeitos os Medicamentos, as Drogas, os Insumos Farmacêuticos e Correlatos, Cosméticos, Saneantes e Outros Produtos, e dá outras Providências. Brasília: Diário Oficial da União. 24 setembro; 1976. p. 12647.

9. Storpirtis S. Princípios de biodisponibilidade, bioequivalência, equivalência farmacêutica e terapêutica de medicamentos. In: Guilhoto LM, Storpirtis S, editors. Atualização terapêutica: o impacto da diversificação de formulações de drogas antiepiléticas na prática clínica. São Paulo: Leitura Médica; 2010. p. 15-44.

10. Brasil. Ministério da Saúde. Agência Nacional de Vigilância Sanitária. Resolução RDC nº 16, de 02 de março de 2007. Regulamento técnico para medicamento similar. Brasília: Diário Oficial da União; 2007.

11. Approved products with therapeutic equivalence evaluations. In: US Department of Health and Human Services PHS, Food and Drug Administration, Center for Drug Evaluation and Research, Office of Pharmaceutical Sciences, Office of Generic Drugs, editor. 33nd ed. Washington, DC2013.

12. Canada. Minister of Health. Health Canada. Comparative Bioavailability Standards: Formulations Used for Systemic Effects. Ottawa, ON2012. p. 11.

13. Neutel JM, Smith DH. A randomized crossover study to compare the efficacy and tolerability of Barr warfarin sodium to the currently available Coumadin®. CVR&R. 1998:49-59.

14. Weibert RT, Yeager BF, Wittkowsky AK, Bussey HI, Wilson DB, Godwin JE, et al. A randomized, crossover comparison of warfarin products in the treatment of chronic atrial fibrillation. The Annals of pharmacotherapy. 2000 Sep;34(9):981-8. PubMed PMID: 10981241. Epub 2000/09/12. eng.

15. Pereira JA, Holbrook AM, Dolovich L, Goldsmith C, Thabane L, Douketis JD, et al. Are brand-name and generic warfarin interchangeable? Multiple n-of-1 randomized, crossover trials. The Annals of pharmacotherapy. 2005 Jul-Aug;39(7-8):1188-93. PubMed PMID: 15914517. Epub 2005/05/26. eng.

16. Lee HL, Kan CD, Yang YJ. Efficacy and tolerability of the switch from a branded to a generic warfarin sodium product: an observer-blinded, randomized, crossover study. Clinical therapeutics. 2005 Mar;27(3):309-19. PubMed PMID: 15878384. Epub 2005/05/10. eng.

17. Henderson JD, Esham RH. Generic substitution: issues for problematic drugs. Southern medical journal. 2001 Jan;94(1):16-21. PubMed PMID: 11213935. Epub 2001/02/24. eng.

18. Paveliu MS, Bengea S, Paveliu FS. Generic Substitution Issues: Brand-generic Substitution, Generic-generic Substitution, and Generic Substitution of Narrow Therapeutic Index (NTI)/Critical Dose Drugs. Maedica. 2011 Jan;6(1):52-8. PubMed PMID: 21977191. Pubmed Central PMCID: PMC3150029. Epub 2011/10/07. eng.

19. Richton-Hewett S, Foster E, Apstein CS. Medical and economic consequences of a blinded oral anticoagulant brand change at a municipal hospital. Archives of internal medicine. 1988 Apr;148(4):806-8. PubMed PMID: 3355300. Epub 1988/04/01. eng.

20. Swenson CN, Fundak G. Observational cohort study of switching warfarin sodium products in a managed care organization. American journal of health-system pharmacy : AJHP : official journal of the American Society of Health-System Pharmacists. 2000 Mar 1;57(5):452-5. PubMed PMID: 10711525. Epub 2000/03/11. eng.

21. Witt DM, Tillman DJ, Evans CM, Plotkin TV, Sadler MA. Evaluation of the clinical and economic impact of a brand name-to-generic warfarin sodium conversion program. Pharmacotherapy. 2003 Mar;23(3):360-8. PubMed PMID: 12627935. Epub 2003/03/12. eng.

22. Leite FQ. Intercambialidade entre medicamentos genéricos: estudo de caso fluoxetina. São Paulo: Universidade Federal de São Paulo. Escola Paulista de Medicina.; 2013.

23. Leiria TL, Pellanda L, Miglioranza MH, Sant'anna RT, Becker LS, Magalhaes E, et al. [Warfarin and phenprocoumon: experience of an outpatient anticoagulation clinic]. Arq Bras Cardiol. 2010 Jan;94(1):41-5. PubMed PMID: 20414525. Epub 2010/04/24. Varfarina e femprocumona: experiencia de um ambulatorio de anticoagulacao. por.

24. Zimerman LI, Fenelon G, Martinelli Filho M, Grupi C, Atié J, Lorga Filho A, et al. Diretrizes Brasileiras de Fibrilação Atrial. Arq Bras Cardiol. 2009;92(6 supl. 1):1-39.

25. Cheng A, Kumar K. Overview of atrial fibrillation2013 May 29, 2013.; 2013(30 June). Available from: http://www.uptodate.com/contents/overview-of-atrial-fibrillation?topicKey=CARD%2F1022&elapsedTimeMs=0&source=search_result&searchTerm=atrial+fibrillation&selectedTitle=1~150&view=print&displayedView=full.

26. Fuster V, Ryden LE, Cannom DS, Crijns HJ, Curtis AB, Ellenbogen KA, et al. ACC/AHA/ESC 2006 Guidelines for the Management of Patients with Atrial Fibrillation: a report of the American College of Cardiology/American Heart Association Task Force on Practice Guidelines and the European Society of Cardiology Committee for Practice Guidelines (Writing Committee to Revise the 2001 Guidelines for the Management of Patients With Atrial Fibrillation): developed in collaboration with the European Heart Rhythm Association and the Heart Rhythm Society. Circulation. 2006 Aug 15;114(7):e257-354. PubMed PMID: 16908781. Epub 2006/08/16. eng.

27. Singer DE, Chang Y, Fang MC, Borowsky LH, Pomernacki NK, Udaltsova N, et al. Should patient characteristics influence target anticoagulation intensity for stroke prevention in nonvalvular atrial fibrillation?: the ATRIA study. Circulation Cardiovascular quality and outcomes. 2009 Jul;2(4):297-304. PubMed PMID: 20031854. Pubmed Central PMCID: PMC2801892. Epub 2009/12/25. eng.

28. Go AS, Mozaffarian D, Roger VL, Benjamin EJ, Berry JD, Borden WB, et al. Heart disease and stroke statistics--2013 update: a report from the American Heart Association. Circulation. 2013 Jan 1;127(1):e6-e245. PubMed PMID: 23239837. Epub 2012/12/15. eng.

29. Camm AJ, Lip GY, De Caterina R, Savelieva I, Atar D, Hohnloser SH, et al. 2012 focused update of the ESC Guidelines for the management of atrial fibrillation: an update of the 2010 ESC Guidelines for the management of atrial fibrillation. Developed with the special contribution of the European Heart Rhythm Association. European heart journal. 2012 Nov;33(21):2719-47. PubMed PMID: 22922413. Epub 2012/08/28. eng.

30. Lorga Filho AM, Azmus AD, Soeiro AM, Quadros AS, Avezum Junior A, Marques AC, et al. Diretrizes brasileiras de antiagregantes plaquetários e anticoagulantes em cardiologia. Arq Bras Cardiol. 2013 Setembro 2013;101(3 supl. 3):1-93.

31. Lip GY, Nieuwlaat R, Pisters R, Lane DA, Crijns HJ. Refining clinical risk stratification for predicting stroke and thromboembolism in atrial fibrillation using a novel risk factor-based approach: the euro heart survey on atrial fibrillation. Chest. 2010 Feb;137(2):263-72. PubMed PMID: 19762550. Epub 2009/09/19. eng.

32. Manning WJ, Singer DE, Lip GY. Antithrombotic therapy to prevent embolization in atrial fibrillation2013 30 June 2013. Available from: www.uptodate.com/contents/antithrombotic-therapy-to-prevent-embolization-in-atrial-fibrillation?source=see_link.

33. Hardman JG LL, Gilman AG. Anticoagulantes, trombolíticos e fármacos antiplaquetários. Goodman & Gilman, As bases farmacológicas da terapêutica. 10 ed. Rio de Janeiro: McGraw-Hill; 2005. p. 1141-58.

34. Marevan® varfarina sódica. Rio de Janeiro, RJ: Farmoquímica S/A; 2013.

35. Riley RS, Rowe D, Fisher LM. Clinical utilization of the international normalized ratio (INR). Journal of clinical laboratory analysis. 2000;14(3):101-14. PubMed PMID: 10797608. Epub 2000/05/08. eng.

36. Connolly SJ, Pogue J, Eikelboom J, Flaker G, Commerford P, Franzosi MG, et al. Benefit of oral anticoagulant over antiplatelet therapy in atrial fibrillation depends on the quality of international normalized ratio control achieved by centers and countries as measured by time in therapeutic range. Circulation. 2008 Nov 11;118(20):2029-37. PubMed PMID: 18955670. Epub 2008/10/29. eng.

37. Micromedex® 2.0.

38. Storpirtis S, Gonçalves JE, Chiann C, Gai MN. Biofarmacotécnica. Rio de Janeiro: Guanabara Koogan; 2011. 321 p.

39. Rosendaal FR, Cannegieter SC, van der Meer FJ, Briet E. A method to determine the optimal intensity of oral anticoagulant therapy. Thrombosis and haemostasis. 1993 Mar 1;69(3):236-9. PubMed PMID: 8470047. Epub 1993/03/01. eng.

40. Petersen P, Boysen G, Godtfredsen J, Andersen ED, Andersen B. Placebo-controlled, randomised trial of warfarin and aspirin for prevention of thromboembolic complications in chronic atrial fibrillation. The Copenhagen AFASAK study. Lancet. 1989 Jan 28;1(8631):175-9. PubMed PMID: 2563096. Epub 1989/01/28. eng.

41. Pocock SJ. The size of a clinical trial. Clinical trials: A practical approach: John Wiley & Sons; 1995. p. 266.

42. Warfarin Sodium. In: DrugPoints® System (electronic version). [Internet]. [cited 17 Julho 2013]. Available from: http://www.micromedexsolutions.com/.

ANEXO I

TERMO DE CONSENTIMENTO LIVRE E ESCLARECIDO

Título da Pesquisa: Equivalência terapêutica entre a varfarina sódica de referência e as genéricas em pacientes adultos com fibrilação atrial no Brasil - ensaio clínico crossover randomizado

O(a) Sr(a). está convidado a participar como voluntário nesta pesquisa que pretende comprovar que o efeito do medicamento genérico varfarina é equivalente ao do medicamento de marca (Marevan®).

***1.* Objetivos**:

Nesta pesquisa verificaremos se a substituição da varfarina de marca (Marevan®) pela genérica pode causar alguma alteração importante nos exames laboratoriais de coagulação ou alterar o número de ocorrências de coágulos indesejáveis (eventos tromboembólicos) e sangramentos observados nos pacientes.

***2.* Procedimentos e envolvimento dos voluntários na pesquisa**:

Participando desta pesquisa será necessário que você retorne duas vezes por mês ao ambulatório da disciplina de Cardiologia da UNIFESP durante os 4 meses da pesquisa para: retirada da medicação, realização dos exames de coagulação (o tempo de protrombina, TP, e a razão normalizada internacional, RNI) e consulta de acompanhamento. Também será solicitado que você evite iniciar qualquer outro tratamento (com medicamentos prescritos por outros médicos, automedicação ou uso de chás e ervas) sem informar o pesquisador do estudo, pois isto atrapalhará a pesquisa.

Normalmente a varfarina não é oferecida aos pacientes do ambulatório, mas participando desta pesquisa você receberá este medicamento no próprio ambulatório. Todos os participantes receberão tanto o medicamento de marca (Marevan®), quanto os dois genéricos (fabricados pelos laboratórios Teuto Brasileiro e União Química Farmacêutica Nacional), porém nem você, nem os médicos ou a farmacêutica saberão quando você receberá cada um deles, pois os comprimidos são semelhantes entre si e as embalagens serão iguais. Esse segredo é fundamental para que o estudo tenha resultados confiáveis.

***3.* Riscos e desconforto**:

Qualquer paciente que utilize o medicamento varfarina corre o risco de sangramentos, que podem ser pequenos (sangramento do nariz, da gengiva, sangue nas fezes), mas também graves hemorragias (hemorragia dentro do crânio, por exemplo) que podem exigir internação ou até causar a morte. Para diminuir o risco de isso acontecer, é necessária a realização do exame de coagulação para ajuste de dose da medicação. Este exame pode causar desconforto passageiro devido à necessidade de coleta de sangue da veia do braço.

Como nesta pesquisa será utilizado o tratamento tradicional, o risco esperado de sangramentos ou de formação de coágulos não é muito diferente daquele que todos os pacientes correm, ou seja, um risco médio. Ainda assim pode ser que a substituição do medicamento de marca pelo genérico aumente minimamente esse risco.

***4.* Benefícios**

Ao final desta pesquisa você poderá descobrir se, na sua saúde, o efeito da varfarina genérica é o mesmo que o do medicamento de marca. Isso será importante, pois assim você poderá ter confiança para adquirir o medicamento genérico, que é mais barato que o de marca. As informações desta pesquisa também poderão ser generalizadas para outros pacientes semelhantes a você, gerando benefícios a eles também.

***5.* Confidencialidade**:

As informações obtidas nesta pesquisa serão analisadas em conjunto com as de outros voluntários, não sendo divulgada a identificação de nenhum paciente. Os dados e o material coletado servirão somente para esta pesquisa.

Rubrica do Participante da Pesquisa	Rubrica do Pesquisador

***6.* Pagamento**

Não será oferecido pagamento aos participantes do estudo, mas também não haverá despesas pessoais para os voluntários, incluindo exames, medicamentos, consultas e transporte.

***7.* Direitos**

Caso você não deseje participar da pesquisa, ou deseje desistir e abandonar a pesquisa a qualquer momento, não haverá nenhum prejuízo para o seu tratamento, sendo garantido o atendimento convencional do ambulatório. Em caso de dano pessoal, diretamente causado pelos procedimentos ou tratamentos propostos neste estudo, você terá direito a tratamento médico neste ambulatório nos moldes de atenção secundária; se houver necessidade de cuidados terciários, estes serão feitos no Hospital São Paulo, da UNIFESP.

***8.* Contato**

Você tem garantido acesso aos profissionais responsáveis pela pesquisa para esclarecimento de eventuais dúvidas. A pesquisadora principal é a farmacêutica Carolina Gomes Freitas, que pode ser contatada pelo telefone celular 9866-8732 ou encontrada na Universidade Federal de São Paulo (UNIFESP), rua Botucatu, 740, 3º andar, sala da PgMIT (Pós-graduação em Medicina Interna e Terapêutica da UNIFESP), bairro Vila Clementino em São Paulo/SP. Se você tiver alguma consideração ou dúvida sobre a ética da pesquisa, entre em contato com o Comitê de Ética em Pesquisa (CEP) – Rua Botucatu, 572 – 1º andar – cj 14, telefone 5571-1062, FAX: 5539-7162 – E-mail:cepunifesp@unifesp.br.

***9.* Consentimento livre e esclarecido**

Declaro que, após convenientemente esclarecido pelo pesquisador e ter entendido o que me foi explicado, consinto em participar do presente Projeto de Pesquisa. Este termo foi elaborado em duas vias que deverão ser assinadas, ficando uma com o(a) Sr(a). e a outra conosco. Todas as folhas deste documento são numeradas e deverão ser rubricadas pelo (a) Sr(a). e pelo pesquisador.

**Identificação do sujeito da pesquisa**		
Nome:		
RG:	Sexo: masculino ( ) feminino ( )	
Data de Nascimento:		Telefone:
Endereço:		

____

Assinatura do Participante da Pesquisa

____

Assinatura da Testemunha (para caso de voluntários analfabetos)

Declaro cumprir todas as exigências descritas neste termo.

____

Assinatura do Pesquisador e RG Local: Data / /

ANEXO II

Interações da varfarina sódica com fármacos, de acordo com a gravidade (desconhecida - *unknown*, pequena – *minor*, moderada – *moderate*, importante – *major*, contraindicada - *contraindicated*). DrugPoint® Summary (42)

Contraindicada

Tamoxifeno, citrato de

Importante

abciximabe	cefpodoxima	dipiridamol	itraconazol	paroxetina	tinzaparina
acenocumarol	ceftazidima	doxorrubicina	latamoxefe	penicilina G	tirofibana
ácido acetilsalicílico	ceftibuteno	dronedarona	leflunomida	penicilina V benzatina	torasemida
ácido nalidíxico	ceftidinir	enoxacino	lepirudina	pentosana, polissulfato sódico de	vacina vírus influenza inativado
ácido valpróico	ceftizoxima	enoxaparina	levofloxacino	piperacilina	Venlafaxina
alefacepte	celecoxibe	eptifibatida	Lycium	posaconazol	vilazodona
Alho	cetoconazol	eritromicina, acistrato de	Marijuana	prasugrel	vincristina
amiodarona	cetoprofeno	erlotinibe	meticilina	procarbazina	vindesina
amoxicilina	ciclofosfamida	Erva de São João / hipérico / hipericão	metotrexato	proguanil	voriconazol
ampicilina tri-hidratada	ciprofloxacino	escitalopram	metronidazol	rivaroxabana	
apixabana	citalopram	etoposídeo	miconazol	Romã	
aprepitanto	claritromicina	etravirina	milnaciprana	ropinirol	
azitromicina	clopidogrel	femprocumona	moxifloxacino	roxitromicina	
bivalirudina	cloxacilina benzatina	fenindiona	nafcilina	salicilato de metila	
Camomila	Cranberry	fenofibrato	nandrolona	sertralina	
capecitabina	dabigatrana	fluconazol	naproxeno	sinvastatina	
carbenicilina dissódica	dabrafenibe	fluoruracila	norfloxacino	sitaxentana	
carboplatina	dalteparina	fluoxetina	noscapina	sulfafurazol	
cefadroxila	danaparoide	fluvoxamina	ofloxacino	sulfametoxazol	
cefalexina	Danshen / tan shen	gatifloxacino	Óleo de peixe	telitromicina	
cefalotina sódica	dapsona	gemifloxacino	oseltamivir	teriflunomida	
cefapirina	deferasirox	Ginkgo biloba	oxacilina	testosterona	
cefepima	desvenlafaxina	imatinibe	oxandrolona	ticarcilina	
cefotaxima	dicloxacilina	infliximabe	papaína	ticlopidina	

Moderada

Abacate	argatrobana	carbamazepina	colesevelam	doxiciclina cálcica	fosamprenavir
Abóbora	armodafinila	carprofeno	colestiramina	duloxetina	fosaprepitanto
Acarbose	Arnica	Cassis	condroitina	Escutelária	Fucus vesiculosus
acemetacina	Assafétida	Castanha-da-índia	contraceptivos orais	esomeprazol	gefitinibe
acetato de metilprednisolona	Astragalus	Cebola	cortisona	espironolactona	gencitabina
ácido etacrínico	atazanavir	cefamandol	Cravo-da-índia	estanozolol	genfibrozila
ácido flufenâmico	atenolol	cefazolina sódica	Cumaru	Etanol	Gengibre
ácido mefenâmico	atovaquona	cefoperazona	Curcumina	etclorvinol	Ginseng
ácido tiaprofênico	azapropazona	cefotetana	danazol	eterobarbe	glibenclamida
Agrimônia / eupatória	azatioprina	ceftriaxona	darunavir	etodolaco	glimepirida
Agripalma	Azevinho	cetorolaco trometamina	delavirdina	etotoína	glipizida
Aipo / salsão	benoxaprofeno	chá preto	demeclociclina	etretinato	glucagon
Álamo / choupo	benzbromarona	chá verde	Dente-de-leão	exenatida	glucosamina
Alcaçuz	betametasona	Chaparral	desipramina	ezetimiba	glutetimida
alclofenaco	bicalutamida	Chorão / salgueiro	Devil's claw	felbamato	Goma guggul
Alface selvagem	Black haw	ciclosporina	dexametasona	fembufeno	griseofulvina
Alfafa	boceprevir	cimetidina	dexlansoprazol	fenilbutazona	halotano
Almíscar-doce / aspérula	Bogbean	Cimicífuga	dextropropoxifeno	fenitoína	heparina cálcica
Aloe	Boldo	Cinchona / quina	diazóxido	fenobarbital	heptabarbe
alopurinol	Borragem	cisaprida	diclofenaco	Feno-grego	hexobarbital
aminoglutetimida	bosentana	cisplatina	dietilestilbestrol	fenoprofeno	hidrato de cloral
amitriptilina	bromelaína	clofibrato	diflunisal	floctafenina	hidrocortisona
amobarbital sódico	bronfenaco	clomipramina	dipirona	flosequinana	ibritumomabe
amoxapina	Buchu	cloranfenicol	disopiramida	fludrocortisona, acetato	ibuprofeno
amprenavir	bufexamaco	clordiazepóxido	dissulfiram	fluoximesterona	ifosfamida
Angélica	butalbital	clorotrianiseno	Dong quai	flurbiprofeno	imipramina, cloridrato
Anis / erva-doce	Canela-da-china	clorpromazina, cloridrato de	dosulepina	flutamida	indometacina
aprobarbital	capsaicina	coenzima Q10	doxepina	fluvastatina	indoprofeno

Ipê roxo / pau d'arco	Milfolhas	piroxicam	rifampicina	tenidape sódico	zafirlucaste
ipriflavona	minociclina	pirprofeno	rifapentina	tenoxicam	zileutona
isoniazida	Mirtilo	Pólen	rifaximina	terbinafina	zomepiraco sódico
isoxicam	mitotano	policosanol	rilonacept	tetraciclina	zotepina
ivacaftor	moracizina	prednisolona	ritonavir	tiamazol/metimazol	
ivermectina	nabumetona	prednisona	rofecoxibe	tibolona	
Kava kava	nelfinavir	Prickly ash	romidepsin	ticlopidina	
lactulose	neomicina	primidona	rosuvastatina	ticlopidina	
lansoprazol	nevirapina	propafenona, cloridrato de	salicilato de bismuto	tigeciclina	
levamisol	niacina	propanolol	salicilatos	tinidazol	
levotiroxina	nilutamida	propifenazona	Salsa / salsinha	tocilizumabe	
lopinavir	nimesulina	propiltiouracila	saquinavir	tocoferol/vitamina E	
lornoxicam	nortriptilina, cloridrato	proquazona	Sarsaparilla, German	tolmetina	
lovastatina	Nutrição enteral	Proteína (alimentação rica em)	Saw palmetto	tolterodina	
Maracujá	Óleo de prímula	protriptilina	secbutabarbital	toremifeno	
Meadowsweet	ômega 3, ésteres etílicos	Quassia	secobarbital	tramadol	
meclofenamato	omeprazol	quetiapina	Senega	trastuzumabe	
melatonina	orlistate	quinestrol	Soja	treprostinila	
meloxicam	oxaprozina	quinidina	sorafenibe	triancinilona acetonida	
mentol	oxifembutazona	quinina	sucralfato	trimipramina	
mercaptopurina	oximetolona	quitosana	sulfassalazina	Unha de gato	
mesalazina	oxitetraciclina	rabeprazol sódico	sulfimpirazona	Urtiga	
mesna	pantoprazol	Raiz forte	sulindaco	valdecoxibe	
metandienona	paracetamol	Raiz-amarela / raiz-laranja / hidraste	sulofenur	vancomicina	
metilfenidato	parametasona	raloxifeno	suprofeno	vemurafenibe	
metilfenobarbital	pentoxifilina	ranitidina	Tamarindo	verinostate	
metiltestosterona	piracetam	retinol / vitamina A	teduglutide	Vitamina K	
mifepristona	pirazolaco	rifabutina	telaprevir	Wintergreen	
