# Supplementary material for: Examining therapeutic equivalence between branded and generic warfarin in Brazil: The WARFA crossover randomized controlled trial
Source: PLoS One. 2021 Apr 1;16(4):e0248567. doi: 10.1371/journal.pone.0248567 (PMC8016229; doi:10.1371/journal.pone.0248567)
Supplement: Attachment — Submitted filename: comments.docx [file pone.0248567.s025.docx]

Comments to the Author 
Thank you for the opportunity to review your article.

The authors report therapeutic equivalence between branded and generic warfarin in a therapeutic equivalence between branded and generic warfarin. Despite the work strengths, I recommend addressing the following comments: 

1. Did you monitor the concentration of branded and generic warfarin in every period?

2. **S4 Table** showed the clinical events in the WARFA trial, and could you record which period these clinical events first occur in? I think this table should be shown in the result section.
